# Bridging the Gap between Genes and Language Deficits in Schizophrenia: An Oscillopathic Approach

**DOI:** 10.3389/fnhum.2016.00422

**Published:** 2016-08-23

**Authors:** Elliot Murphy, Antonio Benítez-Burraco

**Affiliations:** ^1^Division of Psychology and Language Sciences, University College LondonLondon, UK; ^2^Department of Philology, University of HuelvaHuelva, Spain

**Keywords:** neural oscillations, schizophrenia, dynome, genome, oscillopathy, language evolution

## Abstract

Schizophrenia is characterized by marked language deficits, but it is not clear how these deficits arise from the alteration of genes related to the disease. The goal of this paper is to aid the bridging of the gap between genes and schizophrenia and, ultimately, give support to the view that the abnormal presentation of language in this condition is heavily rooted in the evolutionary processes that brought about modern language. To that end we will focus on how the schizophrenic brain processes language and, particularly, on its distinctive oscillatory profile during language processing. Additionally, we will show that candidate genes for schizophrenia are overrepresented among the set of genes that are believed to be important for the evolution of the human faculty of language. These genes crucially include (and are related to) genes involved in brain rhythmicity. We will claim that this translational effort and the links we uncover may help develop an understanding of language evolution, along with the etiology of schizophrenia, its clinical/linguistic profile, and its high prevalence among modern populations.

## Introduction

Schizophrenia is a pervasive neurodevelopmental disorder entailing several (and severe) social and cognitive deficits (van Os and Kapur, 2009). Usually, people with schizophrenia exhibit language problems at all levels, from phonology to pragmatics, which coalesce into problems for speech perception (auditory verbal hallucinations), abnormal speech production (formal thought disorder), and production of abnormal linguistic content (delusions, commonly understood to be distinct from thought disorders), which are the hallmarks of the disease in the domain of language (Stephane et al., 2007, 2014; Bakhshi and Chance, 2015). Importantly, although schizophrenia is commonly defined as a disturbance of thought or selfhood, some authors claim that most of its distinctive symptoms may arise from language dysfunction; in particular, from failures in language-mediated forms of meaning (Hinzen and Rosselló, 2015).

There is ample evidence that schizophrenia is caused by a complex interaction between genetic, epigenetic, and environmental factors. To date, schizophrenia has been related to mutations, copy number variation, or changes in the expression pattern of an extensive number of genes (see O'Tuathaigh et al., 2012; Flint and Munafò, 2014; McCarthy et al., 2014; Schizophrenia Working Group of the Psychiatric Genomics Consortium, 2014 for recent reviews). Many of them point to specific regulatory and signaling pathways (like dopaminergic, glutamatergic, GABAergic, and cholinergic pathways, the neuregulin signaling pathway, and the Akt/GSK-3 pathway) and to specific neural mechanisms (like those involving dendritic spines and synaptic terminals, synapses, gray matter development, and neural plasticity, Buonanno, 2010; Karam et al., 2010; Bennett, 2011; Schizophrenia Working Group of the Psychiatric Genomics Consortium, 2014; Hall et al., 2015). However, the gap between genes, brain abnormalities, and cognitive dysfunction in schizophrenia still remains open, particularly regarding its distinctive linguistic profile.

The goal of this Perspective article is to suggest new ways of bridging the gap between genes and schizophrenia. Cognitive disorders are increasingly being conceived as oscillopathies, or pathological variations of the normal profile of brain rhythmicity (Buzsáki and Watson, 2012; Buzsáki et al., 2013). Current understanding suggests that schizophrenia is characterized by asynchronous neural oscillations, and particularly, by an inhibitory interneuron dysfunction (Moran and Hong, 2011; Pittman-Polletta et al., 2015). Importantly, brain rhythms are heritable components of brain function (Linkenkaer-Hansen et al., 2007), also in pathological conditions (see Hall et al., 2011 for schizophrenia). At the same time, there seems to be a robust link between language disorders and language evolution: recently evolved cognitive abilities are preferably disturbed in disorders because of the reduced resilence of the neural networks (see Benítez-Burraco, 2016 for discussion). The human pattern of brain activity can be conceived of as a slight variation of the patterns observed in other primates (Buzsáki et al., 2013). Accordingly, our species-specific ability to acquire and use languages (aka *language-readiness*) plausibly resulted from the emergence of a new pattern of cortical inhibition and of long-distance connections across the brain (see Boeckx and Benítez-Burraco, 2014a for details), both of which are aspects that are targeted in schizophrenia (Morice and McNicol, 1985; Horn et al., 2012; Jiang et al., 2015). If we are on the right track, we expect that examining language deficits in schizophrenia from this oscillopathic and evolutionary perspective will help us understand its distinctive neurocognitive profile, but also its origins and its prevalence among modern populations.

## From language deficits to the brain in schizophrenia

Schizophrenics have been known to have disordered speech (McKenna and Oh, 2005), but the most severe linguistic changes occur at the internal, conceptual level, where studies frequently examine patients who experience thoughts being “inserted” into them from outside sources or “broadcast” out of their minds and into other people's (Crow, 1980; Frith, 1992). Patients also sometimes hear their thoughts “echoed,” or spoken aloud, and are also known to experience third-person and second-person auditory hallucinations, with an external voice either discussing them or commenting on their actions (Ramsden, 2013, pp. 234–265). Frith and Allen's (1988) review observed “a failure to structure discourse at higher levels.” Abnormalities can also be detected with syntax, however, and this is where we will focus most of our attention. Schizophrenic patients exhibit fewer relative clauses (as their discourse difficulties would predict), shorter utterances, and less clausal embedding (Fraser et al., 1986; Thomas et al., 1987). Importantly, this relative lack of clausal embedding implies that patients do not engage in thoughts about mental states or Theory of Mind (Morice and Ingram, 1982; Morice and McNicol, 1986).

In contrast to normal left-lateralization of activity in fronto-temporal regions during language processing, a wide range of schizophrenic patients exhibit bilateral and right-lateralized activity (Weiss et al., 2005; Diederen et al., 2010). Angrilli et al. (2009) have relatedly proposed that, judging by evoked potentials, certain features of schizophrenia appear to be (partly) a failure of phonological left hemispheric dominance, since the above deficit in lateralization is specific to phonological processing, being absent in semantic and word recognition tasks.

## From brain rhythmicity to language deficits in schizophrenia

Although schizophrenia was for a time deemed “the graveyard of neuropathology” (Plum, 1972) due to its unusually subtle neurophysiological markers, we believe that research in neuronal dynamics (particularly over the past half-decade) has the potential to carve a clearer image of the abnormally-developing brain. Oscillations play a central role in selectively enhancing neural assembly interconnectivity and information processing through the provision of spatio-temporal windows of enhanced or reduced patterns of excitability (Jensen et al., 2014; Weisz et al., 2014), and are consequently strong candidates for the origin of certain cognitive faculties.

If the translational approach taken in Murphy (2015a, 2016) toward the brain dynamics of language is accurate, and if Hinzen and Rosselló (2015) are correct in claiming that linguistic disorganization in schizophrenia “plays a more central role in the pathogenesis of this disease than commonly supposed,” then it is appropriate to inform our understanding of schizophrenia by focusing on the central role of brain rhythms in linguistic computation. If schizophrenia represents a breakdown in normal linguistic cognition, then we would expect to see disruptions in the model of brain dynamics of language processing outlined in Murphy (2015a) when examining the recent, burgeoning literature concerning the oscillatory profile of schizophrenics.

To briefly summarize previous work, it was claimed in Murphy (2015a,b) that set-formation amounts to the α rhythm embedding cross-cortical γ rhythms, with α reflecting long-range cortical interactions (Nunez et al., 2001) and thalamo-cortical loop activity (Nunez and Srinivasan, 2006). The syntactic operation of “Transfer” (which “chunks” constructed objects into short-term memory) was claimed to amount to the embedding of these γ rhythms inside the θ band, generated in the hippocampus. It was also claimed that labeling (maintaining an item in memory before coupling it with another, yielding an independent syntactic identity) amounts to the slowing down of γ to β before β-α coupling, likely involving a basal ganglia-thalamic-cortical loop. These suggestions are in line with Mai et al.'s (2016) finding of γ-related modulations during semantic and syntactic processing (our claims should also not be conflated with the well-known *phonological* oscillatory investigations of Giraud and Poeppel (2012). We will adopt these assumptions here when interpreting the rhythmic literature on schizophrenia.

Since schizophrenia, like other cognitive impairments, appears not to be the result of a locally delimited neural deficit but rather emerges from distributed impairments, neural oscillations, and their role in flexible brain connectivity have recently become the target of research. Investigating the frequency and brain location of the neural oscillations involved in lexical processing in schizophrenia, Xu et al. (2013) conducted an MEG study in which patients discriminated correct from incorrect visually presented stimuli. This lexical decision task revealed that the patients, relative to healthy controls, showed abnormal oscillatory activity during periods of lexical encoding and post-encoding, particularly in the occipital and left frontal-temporal areas (see also Sun et al., 2014). Since a broad range of rhythms were implicated, we will avoid speculation about the specific operations impaired and instead suggest that the results imply familiar problems with semantic memory. However, the results did reveal reduced temporal lobe α and left frontal lobe β activity during lexical processing, suggesting difficulties in assigning lexical classes (labels) to items and successful categorization (findings corroborating the cartographic profile presented above, which included reduced activation during complex sentence processing at left superior frontal cortex). These results corroborate the more general findings of reduced α and β in schizophrenia by Moran and Hong (2011) and Uhlhaas et al. (2008). A level of thalamocortical dysrhythmia was also detected by Schulman et al.'s (2011). MEG study; a discovery which bears on the claim that thalamocortical axons also likely play a role in language externalization (Boeckx and Benítez-Burraco, 2014b). These suggested problems with the mechanisms responsible for phrase structure building also gain support from Ghorashi and Spencer's (2015) findings that attentional load increases β phase-locking factor at frontal, parietal and occipital sites in healthy controls during a visual oddball task but not in schizophrenic patients (although this varied across individuals of different abilities), with the latter group having difficulty attending to and maintaining relevant objects in memory (perhaps as a result of their semantic memory deficits). β-generating circuits may well be responsible, then, for the types of computations attributed to them in Murphy (2015a).

An earlier MEG sentence presentation task by Xu et al. (2012) also found reduced α and β in left temporal-parietal sites, along with reduced δ at left parietal-occipital and right temporal sites, and reduced θ at occipital and right frontal lobe sites, suggesting problems with phrase structure chunking; that is, problems with word movement and phrasal embedding, as attested above (see Ferrarelli et al., 2012). Schizophrenic patients also displayed reduced δ synchrony at left frontal lobe sites after sentence presentation, suggesting semantic processing dysfunctions. These findings are consistent with Hirayasu et al.'s (1998). MRI study of schizophrenic and bipolar individuals, which reported relatively reduced gray matter volumes in the left superior temporal gyrus for schizophrenics. Their results also give some support to the present hypothesis about chunking difficulties in schizophrenia, since they also reported reduced hippocampal volumes. Altogether, these studies are in agreement the findings of Hoffman et al. (1999), who suggested that the core schizophrenic deficit is not centered on attentional-perceptual cognitive processes, but rather verbal working memory (and, hence, difficulties with syntactic computation, given the “chunking” nature of linguistic phrase structure building; see Narita, 2014), mediated by oscillations generated in the hippocampus and left temporal regions (Murphy, 2015a). Başar-Eroğlu et al. (2011) also documented reduced anterior α in response to simple auditory input, suggesting less efficient processing power.

Power and synchrony reductions in evoked γ have also been documented in chronic, first-episode and early-onset schizophrenia (Williams and Boksa, 2010). Given the role of this band in feature binding and object representation (Uhlhaas et al., 2008) and its functional significance in the present model (Murphy, 2015a, 2016), this suggests that schizophrenics have difficulties generating the correct category of semantic objects to employ in successful phrase structure building, as the behavioral results of lexical decision and related tasks appear to verify (likely explaining the features of delusions and formal thought disorder reviewed above). More recent studies appear to support this perspective. The amplitude of EEG γ was measured during phonological, semantic, and visuo-perceptual tasks by Spironelli and Angrilli (2015). Schizophrenic patients, relative to normal controls, exhibited a significantly weaker hemispheric asymmetry across all tasks and reduced frontal γ. Ferrarelli et al. (2008) also found a decreased γ response in schizophrenic patients after TMS stimulation to the frontal cortex, suggesting an impaired ability to efficiently generate this rhythm. This is of particular significance given that γ amplitude has been shown to scale with the number of items held in working memory (Roux et al., 2012), and the limited phrase structure building and syntactic embedding capacities of schizophrenic patients would follow naturally from these results.

Recall also that the model of linguistic computation adopted here invokes a number of cross-frequency coupling operations. It is of interest, then, that schizophrenic patients showed higher γ-α cross-frequency coupling in Popov and Popova's (2015) study of general cognitive performance, despite this co-varying with poorer attention and working memory capacities. The reason for this may be that the increased phase-amplitude-locking likely results in smaller “gamma pockets” of working memory items (as Korotkova et al., 2010 argue on independent grounds) and hence low total γ power. In this instance, the size and order of working memory sequences outputted by the conceptual systems is not optimally compatible with the oscillopathic profile, leading to greater rhythmic excitability, and yet inhibited linguistic functionality. Global rhythmicity is consequently disrupted due to unusually strong fronto-parietal interconnectivity. We believe that this represents a genuine neural mechanism of an “interface” between syntactically generated conceptual representations and external (memory) systems; a highly significant finding if corroborated by further experimental studies.

Corroborating Angrilli et al.'s (2009) above hypothesis about schizophrenia being a failure of left-hemispheric phonological dominance, an MEG study of the oscillatory differences between bipolar disorder and schizophrenia revealed that schizophrenic patients showed delayed phase-locking in response to speech sounds in the left hemisphere, relative to bipolar individuals and normal controls (Oribe et al., 2010). This lack of left-hemispheric dominance may trigger confusion about internal and external voices and bring about a number of delusions, with language's normal computational functioning being derailed. The left hypofrontality documented by Spironelli et al. (2011), with schizophrenic patients showing greater δ amplitudes over language-relevant sites (that is, greater functional inhibition), similarly point to a general functional deficit at the core memory sites of linguistic representations. It is also significant that the role attributed to θ in the present dynomic model gains support from the finding that this rhythm has greater amplitude in left superior temporal cortex during auditory hallucinations in schizophrenia (Ishii et al., 2000), as opposed to steady θ during resting state, with patients being seemingly incapable of regulating chunking operations. Given the identification of such dysrhythmias in schizophrenia, repetitive TMS (rTMS) could be used as a therapeutic intervention to modulate the oscillations responsible for the abnormal linguistic profile documented above, as has been done to improve performance on visual tasks (Farzan et al., 2012; Barr et al., 2013). The oscillopathic profile constructed here is presented in Table 1.

**Table 1:** **Summary of the present cognome-dynome model of linguistic computation and the observed differences in schizophrenia, where “cognomen” refers to the operations available to the human nervous system (Poeppel, 2012) and “dynome” refers to brain dynamics (Kopell et al., 2014); lSTG denotes left superior temporal gyrus, AVH denotes auditory verbal hallucination**.

**Frequency band**	**Role in the present model of language computation**	**Observed and predicted differences in schizophrenia**
Delta (~0.5–4 Hz)	Involved in phrasal processing and possibly labeling.	Reduced at left parietal-occipital sites during sentence processing; predicted to be disrupted in processing phrasal embedding and relative clauses.
Theta (~4–10 Hz)	Hippocampal source; embeds γ to generate cyclic transfer of syntactic objects; involved more generally in memory retrieval.	Reduced at occipital and frontal lobe sites during sentence processing; increased at lSTG during AVHs; predicted to be reduced in deictic and definite NPs.
Alpha (~8–12 Hz)	Synchronizes distant cortical regions; embeds γ generated cross-cortically to yield inter-modular set-formation; involved in lexical decision making.	Reduced at left temporal lobe during lexical and sentence processing; predicted to be disrupted during certain lexicalisations.
Beta (~10–30 Hz)	When γ is slowed to β and coupled with α via a basal ganglia-thalamic-cortical loop, syntactic objects are labeled; holds objects in memory.	Reduced at left frontal lobe during lexical processing; predicted to be disrupted in the maintenance of syntactic objects in embedded clauses.
Gamma (~30–100 Hz)	Generates syntactic objects before β holds them in memory; central role in a number of linguistic operations; involved in lexical processing.	Reduced at frontal sites during semantic tasks; higher cross-frequency coupling with occipital α; predicted to be disrupted in language-related memory tasks.

## Schizophrenia-related genes and some evolutionary concerns

As noted in the introduction, the number of genes related to schizophrenia has been growing over recent years. Interestingly, some of them are involved in the maintenance of the adequate balance between neuronal excitation and inhibition and/or have been related to language dysfunction. Likewise, as we also noted above and will discuss in detail below, a robust link exists between evolution and abnormal development and, in particular, between language evolution and schizophrenia. In this section we focus on candidate genes for schizophrenia that are involved in brain rhythmicity and that have been related to language impairment or to the dysfunction of basic cognitive abilities involved in language processing, but also on genes important for language evolution that play a role in brain rhythmicity and that are candidates for schizophrenia. The genes we highlight seem to us robust candidates for language deficits in this condition.

### Schizophrenia-candidates and brain rhythmicity

Among the genes related to schizophrenia that play a role in brain oscillations and that have been associated to language dysfunction one finds *ZNF804A*. This gene encodes a zinc finger binding protein important for cortical functioning and neural connectivity, involved in growth cone function and neurite elongation (Hinna et al., 2015). GWAs analyses have identified a SNP tagging an intronic region of the gene (Gurung and Prata, 2015) which have been found to impact on white matter microstructure (Mallas et al., 2016). Schizophrenia risk polymorphisms of *ZNF804A* have been also related to differences in performance in the domain of phonology, such as in reading and spelling tasks (Becker et al., 2012), but also in the domain of semantics, specifically in task evaluating category fluency (Nicodemus et al., 2014). ZNF804A modulates hippocampal γ oscillations and, ultimately, the co-ordination of distributed networks belonging to the hippocampus and the prefrontal cortex (Cousijn et al., 2015), which are aspects known to be impaired in schizophrenia, as noted above (Uhlhaas et al., 2008; Godsil et al., 2013). Likewise, both NRG1 and its receptor ERBB4, which have been posited as promising candidates for schizophrenia as resulting from next-generation sequencing analyses (Agim et al., 2013; Hatzimanolis et al., 2013), enhance synchronized oscillations of neurons in the prefrontal cortex, known to be reduced in schizophrenia, via inhibitory synapses (Fisahn et al., 2009; Hou et al., 2014). Specifically NRG1 increases the synchrony of pyramidal neurons via presynaptic interneurons and the synchrony between pairs of interneurons through their mutually-inhibitory synapses (Hou et al., 2014). Risk polymorphisms of *NRG1* are associated with increased IQs as well as memory and learning performance, along with language in subjects with bipolar disorder (Rolstad et al., 2015). Moreover, risk alleles for the gene correlate with reduced left superior temporal gyrus volumes (a robust imaging finding in schizophrenia, Tosato et al., 2012), a region related to language abilities (Aeby et al., 2013). Another gene of interest is *PDGFR*, which encodes the β subunit of the platelet-derived growth factor (PDGF) receptor, known to be involved in the development of the central nervous system. *Pdgfr-*β knocked-out mice show reduced auditory phase-locked γ oscillations, which correlates with anatomical (e.g., reduced density of GABAergic neurons in the amygdala, hippocampus, and medial prefrontal cortex), physiological (alterations of prepulse inhibition) and behavioral (reduced social behavior, impaired spatial memory and problems with conditioning) hallmarks of schizophrenia (Nguyen et al., 2011; Nakamura et al., 2015). Additional evidence of the involvement of this gene in schizophrenia comes from risk polymorphisms analyses (Kim et al., 2008). Interestingly, *PDGFRA* has been found to act downstream of *FOXP2*, the renowned “language gene,” to promote neuronal differentiation (Chiu et al., 2014, more on *FOXP2* below).

Other genes of interest encode ion channels. Genome-wide analyses (GWAs) have identified the schizophrenia risk gene *CACNA1I* as one of the genes that may contribute to sleep spindle deficits (Manoach et al., 2015). Sleep spindles are a type of brain rhythm that recurs during non-rapid eye movement sleep and that constrains aspects of the thalamocortical crosstalk, impacting on sensory transmission, cortical plasticity, memory consolidation, and learning (Manoach et al., 2015). *CACNA1I* encodes a calcium channel and is abundantly expressed in the spindle generator of the thalamus. Likewise *CACNA1C* encodes the alpha 1C (α1C) subunit of the Cav1.2 voltage-dependent L-type calcium channel, a calcium channel involved in the generation of β to γ waves during wakefulness and rapid eye movement (REM) sleep, and ultimately in sleep modulation; all of which are aspects known to be altered in schizophrenics (Kumar et al., 2015). Intriguingly, *CACNA1C* is related to semantic (but not lexical) verbal fluency in healthy individuals; conversely, risk alleles of this gene correlate with lower performance scores, and thus with non-fluent verbal performance of schizophrenics (Krug et al., 2010). Two proteins associated with ion channels are also worth considering, namely DPP10 and CNTNAP2. DPP10 is a membrane protein that binds specific K^+^ channels and modifies their expression and biophysical properties (Djurovic et al., 2010). Also CNTNAP2 is associated with K^+^ voltage-gated channels, particularly, in the axon initial segment of pyramidal cells in the temporal cortex, that are mostly innervated by GABAergic interneurons (Inda et al., 2006). Several studies have correlated *CNTNAP2* with schizophrenia, including CNV and SNPs studies (Friedman et al., 2008; Ji et al., 2013). The gene is also a candidate for several types of language disorders, including child apraxia of speech (Worthey et al., 2013), dyslexia (Peter et al., 2011), SLI (Newbury et al., 2011), language delay, and language impairment (Petrin et al., 2010; Sehested et al., 2010). CNTNAP2 additionally affects language development in the normal population (Whalley et al., 2011; Whitehouse et al., 2011; Kos et al., 2012), apparently because of its effects on brain connectivity and cerebral morphology (Scott-Van Zeeland et al., 2010; Tan et al., 2010; Dennis et al., 2011) and dendritic arborization and spine development (Anderson et al., 2012). *CNTNAP2* is also a target of FOXP2 (Vernes et al., 2008).

Several genes encoding neurotransmitter receptors have been also related to both abnormal brain oscillation patterns and language deficits in schizophrenia. *HTR1A* encodes the receptor 1A of serotonin and modulates hippocampal γ oscillations, seemingly impacting on behavioral and cognitive functions, such as learning and memory linked to serotonin function (Johnston et al., 2014). Several studies involving common polymorphisms of this gene highlight *HTR1A* as a promising candidate for schizophrenia risk, treatment response to the disease, and cognitive dysfunction in this condition (Gu et al., 2013; Lin et al., 2015; Takekita et al., 2015). Similarly, receptors of NMDA, particularly those containing the subunit NR2A, encoded by *GRIN2A*, are known to be reduced in fast-firing interneurons in schizophrenics, which plays a critical role in γ oscillation formation; a blockade of NR2A-containing receptors gives rise to strong increases in γ power and a reduction in low-frequency γ modulation (Kocsis, 2012). More generally, functional (GT)n polymorphisms in the promoter of the gene have been associated with the disease (Iwayama-Shigeno et al., 2005; Tang et al., 2006; Liu et al., 2015), and genome-wide association analyses has identified *GRIN2A* as a risk factor for schizophrenia (Lencz and Malhotra, 2015), emerging as a promising candidate because of its expression in the adult neocortex (Ohi et al., 2016). Additionally, mutations in *GRIN2A* cause epilepsy-aphasia spectrum disorders, including Landau-Kleffner syndrome and continuous spike and waves during slow-wave sleep syndrome (CSWSS), in which speech impairment and language regression are prominent symptoms (Carvill et al., 2013; Lesca et al., 2013). The gene has been related as well to rolandic epilepsies, the most frequent epilepsies in childhood, in which cognitive, speech, language, and reading problems are commonly observed (Dimassi et al., 2014). Speech problems linked to *GRIN2A* mutations include imprecise articulation, impaired pitch and prosody, and hypernasality, as well as poor performance on maximum vowel duration and repetition of monosyllables and trisyllables, resulting in lifelong dysarthria and dyspraxia (Turner et al., 2015). Finally, cannabinoid-1receptor, encoded by *CNR1*, modulates θ and γ oscillations in several areas of the brain, including the hippocampus, impacting on sensory gating function in the limbic circuitry (Hajós et al., 2008). CNR1-positive GABA-ergic interneurons have been also involved in several aspects of behavior, including response to auditory cues (Brown et al., 2014). Translational convergent functional genomics studies have highlighted CNR1 as an important gene for schizophrenia onset (Ayalew et al., 2012). Several risk polymorphisms of the gene have been related to the disease, and specifically, to brain changes and metabolic disturbances in schizophrenics (Yu et al., 2013; Suárez-Pinilla et al., 2015). Interestingly, *CNR1* has also been linked to cases of complete absence of expressive speech (Poot et al., 2009). *CNR1* is functionally linked to the last gene we wish to highlight, namely, *DISC1* (Xie et al., 2015). *DISC1* encodes a protein involved in neurite outgrowth, cortical development and callosal formation (Brandon and Sawa, 2011; Osbun et al., 2011). *DISC1* is a historical candidate for schizophrenia (but also to other cognitive disorders like ASD), although its status as a candidate is controversial, provided that most GWAs and CNV analyses have been unable to independently implicate it in the disease (see Farrell et al., 2015 for discussion; see Ayalew et al., 2012 for a promising result). Nonetheless, in hippocampal area CA1 of a transgenic mouse that expresses a truncated version of *Disc1* mimicking the schizophrenic genotype, θ burst-induced long-term potentiation (and ultimately, long-term synaptic plasticity) has been found altered (Booth et al., 2014). The ability of DISC1 to regulate excitatory-inhibitory synapse formation by cortical interneurons depends on its inhibitory effect on NRG1-induced ERBB4 activation and signaling, ultimately effecting the spiking interneuron-pyramidal neuron circuit (Seshadri et al., 2015). *DISC1* is also a target of FOXP2 (Walker et al., 2012).

### Schizophrenia-candidates and language evolution

As pointed out above, there exists a robust link between evolution and abnormal development. Because, as noted in the introduction, brain rhythms are heritable components of brain function, and because patterns of brain rhythmicity are species-specific and disorder-specific, we hypothesized that new candidates for language dysfunction in schizophrenia under our oscillopathic view may emerge from the examination of candidate genes for the evolution of language-readiness in our species. As we also pointed out in the introduction, our distinctive ability for acquiring and using language has been hypothesized to have resulted from the emergence of new patterns of cortical rhythmic coupling that habilitated the neuronal workspace needed for transcending the boundaries of core knowledge systems and being able to form cross-modular concepts (known to be affected in schizophrenia); in turn, these changes may have resulted from the brain changes linked to the globularization of the anatomically-modern human (AMH) skull (see Boeckx and Benítez-Burraco, 2014a for details). In a series of related papers, we have put forth a list of tentative candidates for globularization and language-readiness (Boeckx and Benítez-Burraco, 2014a,b; Benítez-Burraco and Boeckx, 2015; see Table 2). As discussed there, core candidates for globularization and language readiness fulfill the following criteria: they show (or are functionally related to genes showing) differences with extinct hominin species, particularly, with Neanderthals/Denisovans, which affect their regulatory regions, their coding regions, and/or their methylation patterns; they play some role in brain growth, regionalization, and/or neural interconnection; they have been associated (or are functionally related to genes associated) to conditions in which language, or cognitive abilities important for language, are impaired; and they are candidates (or are functionally related to candidates) for craniosynostosis or some other conditions affecting skull development. Our list of candidates encompasses genes involved in bone development, brain development (specifically of GABAergic neurons), and more generally, brain-skull cross-talk, like *RUNX2*, some DLX genes (including *DLX1, DLX2, DLX5*, and *DLX6*), and some *BMP* genes (like *BMP2* and *BMP7*). It also includes genes that regulate subcortical-cortical axon pathfinding and that are involved in the externalization of language (such as *FOXP2, ROBO1*, and the genes encoding the SLITs factors). Finally, it also comprises genes connecting the former two interactomes, including *AUTS2* and some of its partners. We have found ample evidence, *in silico* and in the available literature, supporting the biological reliability of these interactomes. Moreover, we have collected some empirical evidence suggesting that many of the genes we regard important for language evolution are dysregulated in clinical conditions involving skull, brain, and cognitive anomalies. Accordingly, differential expression of several of our candidates (*DLX5, ROBO1, SLIT2, NCAM1, TGFB2, DCN, RUNX2*, and *SFRP2*) was found *in vivo* in the sutures of people with non-syndromic craniosynostosis, which are prematurely ossified, and also *in vitro* in cells induced toward osteogenic differentiation (Lattanzi et al., 2016).

**Table 2:** **Genes discussed in Section Schizophrenia-related genes and some evolutionary concerns**.

**Gene**	**LR**	**BR**	**SZ**	**Selected references**	**Gene**	**LR**	**BR**	**SZ**	**Selected references**
*ABL1*	+				*KDM5B*	+			
*AKT1*	+	+	++	Emamian et al., 2004; Wockner et al., 2014	*LHX2*	+			
*ANAPC10*	+				*MAPK1*	+	+		
*APOE*	+	+	+	Verbrugghe et al., 2012	*MAPK14*	+	+	++	Onwuameze et al., 2013; Chang et al., 2015
*ARHGAP32*	+				*MECP2*	+	+	++	Cohen et al., 2002; McCarthy et al., 2014
*ARHGEF6*	+				*MEF2A*	+		++	Thygesen et al., 2015
*ARX*	+	+			*MET*	+		++	Burdick et al., 2010
*ASCL1*	+				*NCAM1*	+		++	Vawter et al., 2001; Atz et al., 2007; Ayalew et al., 2012
*ASPM*	+				*NCOA6*	+			
*AUTS2*	+		+	Zhang et al., 2014	*NFASC*	+			
*BAZ2A*	+				*NKX2-1*	+			
*BGLAP*	+				*NODAL*	+			
*BMP2*	+				*NOTCH1*	+			
*BMP7*	+				*NOVA1*	+			
*CACNA1C*		+	++	Krug et al., 2010	*NR1H2*	+			
*CACNA1I*		+	++	Manoach et al., 2015	*NRG1*	+	+	++	Ayalew et al., 2012; Agim et al., 2013; Hatzimanolis et al., 2013
*CBL*	+	+			*NRG3*	+		+	Kao et al., 2010; Hatzimanolis et al., 2013; Zeledón et al., 2015
*CDC42*	+		++	Gilks et al., 2012; Datta et al., 2015	*NTN1*	+			
*CDC42BPB*	+		+	Narayan et al., 2008	*OTX2*	+			
*CDC42EP4*	+		+	Datta et al., 2015	*PAK5*	+			
*CDH1*	+				*PAK6*	+			
*CDKN1A*	+				*PARP1*	+			
*CEBPB*	+				*PAX3*	+			
*CEP192*	+				*PAX6*	+			
*CITED2*	+				*PCDH11*	+			
*CKAP5*	+				*PCM1*	+		+	Kamiya et al., 2008
*CLOCK*	+		+	Zhang et al., 2011; Jung et al., 2014	*PCNT*	+			
*CMIP*	+				*PDGFR*		+	++	Kim et al., 2008
*CNR1*		+	++	Ayalew et al., 2012; Onwuameze et al., 2013	*PDX1*	+			
*CNTNAP2*	+	+	++	Friedman et al., 2008; Ji et al., 2013	*PIN1*	+			
*CREBBP*	+				*PITPNA*	+			
*CTIP2*	+				*PLAUR*	+	+		
*CTNNB1*	+		+	Levchenko et al., 2015	*POU3F2*	+		++	Huang et al., 2005; Potkin et al., 2009
*DCC*	+		+	Grant et al., 2007, 2012	*PQBP1*	+			
*DIP2A*	+				*PTEN*	+	+		
*DISC1*	+	+	++	Johnstone et al., 2011; Ayalew et al., 2012	*PTPRB*	+			
*DISP1*	+				*PVALB*	+			
*DLL1*	+				*RELN*	+		++	Shifman et al., 2008; Ayalew et al., 2012; Li et al., 2015
*DLX1*	+		+	Kromkamp et al., 2003	*ROBO1*	+		++	Potkin et al., 2009, 2010
*DLX2*	+				*ROBO2*	+		++	Potkin et al., 2009, 2010
*DLX5*	+	+	+	Cho et al., 2015	*RUNX1*	+			
*DLX6*	+	+	+	Cho et al., 2015	*RUNX2*	+		+	Benes et al., 2007
*DPP10*		+			*RUNX3*	+			
*DUSP1*	+				*SATB2*	+			
*DYRK1A*	+	+			*SFRP2*	+			
*DYX1C1*	+				*SHH*	+		+	Boyd et al., 2015
*EGFR*	+		+	Benzel et al., 2007	*SIRT1*	+	+	++	Kishi et al., 2011; Wang et al., 2015
*EGR1*	+	+	+	Pérez-Santiago et al., 2012	*SIX3*	+			
*ELAVL2*	+		++	Yamada et al., 2011	*SLIT1*	+			
*ELP4*	+	+			*SLIT2*	+			
*EMX2*	+				*SLITRK3*	+	+		
*EP300*	+				*SMAD9*	+			
*ERBB4*	+	+	++	Agim et al., 2013; Hatzimanolis et al., 2013	*SMURF1*	+			
*ETV4*	+				*SOLH*	+			
*EXOC6B*	+				*SOX10*	+		++	Iwamoto et al., 2005; Drerup et al., 2009; Yuan et al., 2013; Wockner et al., 2014
*FEZF2*	+				*SOX2*	+			
*FGF7*	+				*SOX3*	+			
*FGF8*	+				*SOX9B*	+			
*FGFR1*	+		+	Mor et al., 2013	*SPAG5*	+			
*FLNA*	+	+			*SPC7*	+			
*FMR1*	+	+	+	Kovács et al., 2013; Folsom et al., 2015	*SPP1*	+			
*FOXA1*	+				*SRGAP2*	+	+		
*FOXA2*	+				*SRGAP3*	+		+	Wilson et al., 2011; Waltereit et al., 2012
*FOXG1*	+	+			*SRPX2*	+	+		
*FOXO1*	+				*TBR1*	+			
*FOXP1*	+		++	Ingason et al., 2015	*TGFB*	+		+	Frydecka et al., 2015,
*FOXP2*	+		+	Španiel et al., 2011; Li et al., 2013	*TLE2*	+			
*FRP*	+				*TLE3*	+			
*GAD1*	+	+	++	Ayalew et al., 2012; Mitchell et al., 2015; Lett et al., 2016	*TP53*	+		+	Ni et al., 2005; Molina et al., 2011
*GADD45G*	+				*TSC1*	+	+		
*GBX2*	+				*USF1*	+			
*GLI3*	+				*USH2A*	+			
*GRIN2A*		+	++	Lencz and Malhotra, 2015; Ohi et al., 2016	*VCAM1*	+			
*GTF2I*	+				*VCAN*	+			
*GTF3C3*	+				*VDR*	+			
*HES1*	+				*WNT5A*	+			
*HOXA2*	+				*YAP1*	+			
*HRAS*	+	+			*ZBTB20*	+			
*HTR1A*		+	+	Gu et al., 2013; Lin et al., 2015; Takekita et al., 2015	*ZFHX1B*	+			
*ITGB4*	+				*ZNF804A*		+	++	Becker et al., 2012; Nicodemus et al., 2014
*KATNA1*	+								

The first column contains the official name of the genes according to the Hugo Gene Nomenclature Committee (http://www.genenames.org/). The three remaining columns show whether the genes are candidates for language readiness according to Boeckx and Benítez-Burraco (2014a,b) and Benítez-Burraco and Boeckx (2015) (column 2: LR), are involved in brain rhythmicity according to the available literature, consulted via PubMed (http://www.ncbi.nlm.nih.gov/pubmed)(column 3: BR), or are candidates for schizophrenia (idem.) (column 4: SZ). The last column contains the most relevant papers that are indicative of an association between the gene and the disease. Candidate genes for schizophrenia resulting from GWA and CNV/exome sequencing studies are marked with ++ and should be regarded as more robust candidates than those resulting from candidate gene studies (marked with +) (for further details, see the Supplementary Files).

Interestingly, we have found that candidates for schizophrenia are overrepresented among the genes highlighted by Benítez-Burraco and Boeckx (Table 2). Accordingly, nearly 5% of the human genes are expected to be related to the disease [assuming that the human genome contains about 20,000 protein-coding genes and that about 1000 of them have been associated to schizophrenia, according to the Schizophrenia Gene repository (http://www.szgene.org/)], In turn, around 30% of candidates for language readiness are also candidates for schizophrenia (42 out of 153 in Table 2). Because the involvement of these genes in language development and evolution, this overlapping may account for the observed deficits in schizophrenia regarding language abilities. These genes are discussed in detail in the Supplementary Materials to this paper. Moreover, several of these common candidates for language-readiness and schizophrenia also play a role in brain rhythmicity, including *AKT1, APOE, DLX5, DLX6, EGR1, FMR1, GAD1, MAPK14, MECP2*, and *SIRT1* (Table 2). These genes attracted our attention as promising new candidates for the oscillopathic nature of language deficits in schizophrenia. Finally, some of the candidates for the schizophrenia dynome interact with some of the genes encompassing these interactomes important for our language-readiness (Figure S2). In our opinion, all these findings reinforce the view that language impairment in schizophrenia results from (and can be confidently construed in terms of) abnormal patterns of brain connectivity and dynamics.

This overrepresentation of candidates for schizophrenia among the genes involved in language evolution is an intriguing finding. It has been hypothesized that schizophrenia candidate genes were involved in the evolution of the human brain and that the processes they contributed to improving are identical to those impaired in schizophrenics. For example, the human prefrontal cortex, which is responsible for many human-specific cognitive abilities, is differently organized in humans compared to great apes as a result of a recent reorganization of the frontal cortical circuitry; at the same time, these circuits are impaired in schizophrenia and other psychiatric and neurological conditions (Teffer and Semendeferi, 2012). Nonetheless, when it comes to testing this hypothesis, contradictory results have been obtained. Concerning the protein-coding regions of genes associated to psychiatric disorders Ogawa and Vallender (2014) did not find evidence of differential selection in humans compared to non-human primates, although elevated dN/dS was observed in primates and other large-brained taxa like cetaceans (dN/dS is the average number of nucleotide differences between sequences per non-synonymous site referred to the average number of nucleotide differences between sequences per synonymous site; dN/dS values that are significantly higher than 1 are indicative of positive selection). However, recent analyses based on large GWAs of schizophrenia and data of selective sweeps in the human genome compared to Neanderthals suggest that brain-related genes showing signals of recent positive selection in AMHs are also significantly associated with schizophrenia (Srinivasan et al., 2016), supporting the view that schizophrenia may be a by-product of the changes in the human brain that led to modern cognition. Interestingly, among the loci highlighted by Srinivasan et al. (2016), we have found several genes related to language development, language impairment, and language evolution, which strike us as new promising candidates for language dysfunction in schizophrenia. Among them, we wish highlight: *FOXP1, GATAD2B, MEF2C, NRG3, NRXN1*, and *ZNF804A* (see Supplementary Materials for details). We wish also highlight that some of the genes involved in brain rhythmicity (reviewed above) also show differences in the human lineage. *DPP10* shows signals of differential expression in the human brain compared to primates and sequences at DPP10 show regulatory motifs absent in archaic hominins and signals of strong selection in modern human populations (Shulha et al., 2012). Likewise, DISC1 interacts with PCNT, mentioned by Green et al. (2010) as being amongst the proteins that show non-synonymous and non-fixed changes compared to Neanderthals, and a candidate for dyslexia (Poelmans et al., 2011). Finally, the human CNTNAP2 protein bears a fixed change (I345V) compared to the Denisovan variant (Meyer et al., 2012) and it is related in addition to NFASC, a protein involved in postsynaptic development and neurite outgrowth (Kriebel et al., 2012) which also shows a fixed change (T987A) in AMHs compared to Neanderthals/Denisovans (Pääbo, 2014, Table S1).

Some authors have explicitly linked the aetiopathology of schizophrenia and the evolution of language. According to Arbib and Mundhenk (2005) the primate mirror neurons, which fire both when the animal manipulates an object and when it sees another conspecific manipulating it, provided the scaffolding for imitation abilities involved in language acquisition. At the same time, schizophrenics show a spared ability to generate actions, whether manual or verbal, but they lack the ability to attribute the generation of that action to themselves. More drastically, Crow (1997) suggested that schizophrenia is the “price we paid for language.” According to him, schizophrenia represents an extreme of variation of hemispheric specialization and a single genetic mechanism (involving both the X and Y chromosomes) that was modified during recent human history can account for this variation, because it generates epigenetic diversity related to both the species capacity for language and the predisposition to psychosis (Crow, 2008).

Our findings provide a different causative explanation to the origins and prevalence of schizophrenia, while still supporting the view that the etiopathology of this condition is heavily rooted in the evolution of human cognition. The genes discussed here map onto specific neuronal types (mostly, GABAergic), particular brain areas (several cortical layers, thalamic nuclei), particular physiological processes (the balance between inhibition and excitation), specific developmental processes (inter and interhemispheric axon pathfinding), and particular cognitive abilities (formal thought), all of which are aspects known to be impaired in schizophrenia. At the same time, all of them are involved in language development and processing and many of them have been modified during our recent evolutionary history. Interestingly, schizophrenia associations have been recently proved to be strongly enriched at enhancers that are active in tissues with important immune functions, giving support to the view that immune dysregulation plays a role in schizophrenia (Schizophrenia Working Group of the Psychiatric Genomics Consortium, 2014). Likewise, changes in the brain/immune system crosstalk have been hypothesized to have contributed to the changes in brain connectivity that prompted the emergence of our language-readiness (Benítez-Burraco and Uriagereka, 2015).

Accordingly, instead of thinking of schizophrenia as the “price we paid for language,” we believe a more accurate claim is that schizophrenia is the price we paid for a globular braincase housing more efficient and widespread recursive oscillatory embeddings. Because the more novel a neural network is in evolutionary terms, the less resilient it is (due to its lack of robust compensatory mechanisms, Toro et al., 2010), schizophrenia is found as a high prevalent condition among modern populations. This view is in line with current approaches to the etiology of complex diseases in humans, according to which high prevalent conditions of a multifactorial nature resulted from the de-canalization of the robust primate condition as a consequence of our evolutionary history (involving demographic bottlenecks, specific mutations, and cultural changes that uncovered cryptic variation, see Gibson, 2009 for details).

## Conclusions

The considerations we have made here may provide a suitable response to Dehaene et al.'s (2015, p. 2) observation that linguistic computation requires “a specific recursive neural code, as yet unidentified by electrophysiology, possibly unique to humans, and which may explain the singularity of human language and cognition.” Hierarchical rhythmic coupling operations of the kind proposed in Murphy (2015a, 2016) and discussed here may also provide ways of integrating different forms of hierarchical representations, such as phonological, semantic and syntactic information (see Ding et al., 2016). Disruptions to the present dynomic model of linguistic computation may represent a comprehensive, unifying account of language-related neurocognitive disorders As we have argued, schizophrenia is of particular interest because it represents a mode of cognition and externalization of thought distinct from, but plainly related to, normally functioning linguistic cognition. Importantly, this deviance seems construable in terms of an alteration of the cognome-dynome cross-talk. A dynomic perspective cuts across the traditional positive-negative symptom division, being implicated both in abnormal active processes and in the absence of normal functions. This view is in line with more general, recent moves in neuroscience to view psychiatric illnesses as oscillatory connectomopathies (Cao et al., 2016; Vinogradov and Herman, 2016). At the same time, the considerations we have presented also reinforce the view that the survey of the evolutionary itinerary followed by our faculty of language should help unravel abnormal cognitive/linguistic development in our species (and vice versa). The high number of candidates for schizophrenia selected in our species ostensibly proves this. We further expect that the present proposal has the potential to provide robust endophenotypes of schizophrenia (in the form of specific brain oscillation patterns and novel gene candidates) and contribute to an improved diagnosis and treatment of the disorder.

## Author contributions

EM contributed primarily to Sections From language deficits to the brain in schizophrenia and From brain rhythmicity to language deficits in schizophrenia, AB contributed primarily to Sections Introduction and Schizophrenia-related genes and some evolutionary concerns. Both authors contributed equally to Section Conclusions.

### Conflict of interest statement

The authors declare that the research was conducted in the absence of any commercial or financial relationships that could be construed as a potential conflict of interest. The Review Editor PU and handling Editor AK declared their shared affiliation, and the handling Editor states that the process nevertheless met the standards of a fair and objective review.
