# Supplementary material for: Bridging the Gap between Genes and Language Deficits in Schizophrenia: An Oscillopathic Approach
Source: Front Hum Neurosci. 2016 Aug 23;10:422. doi: 10.3389/fnhum.2016.00422 (PMC4993770; doi:10.3389/fnhum.2016.00422)
Supplement: Supplementary file 1 [file DataSheet1.PDF]

Supplementary Material

Bridging the gap between genes and language deficits in schizophrenia: an oscillopathic approach

Elliot Murphy*, Antonio Benítez-Burraco

*** Correspondence:** elliotmurphy91@gmail.com

Supplementary Data: Functional characterization of candidates for schizophrenia related to language evolution

A. *Candidates genes for schizophrenia that belong (or interact) to the set of genes involved in language evolution (according to Benítez-Burraco and Boeckx)*

As noted in the main text, the set of genes put forth by Benítez-Burraco and Boeckx (Boeckx and Benítez-Burraco 2014a,b, Benítez-Burraco and Boeckx 2015) is clustered around three related putative interactomes (Figure S1).

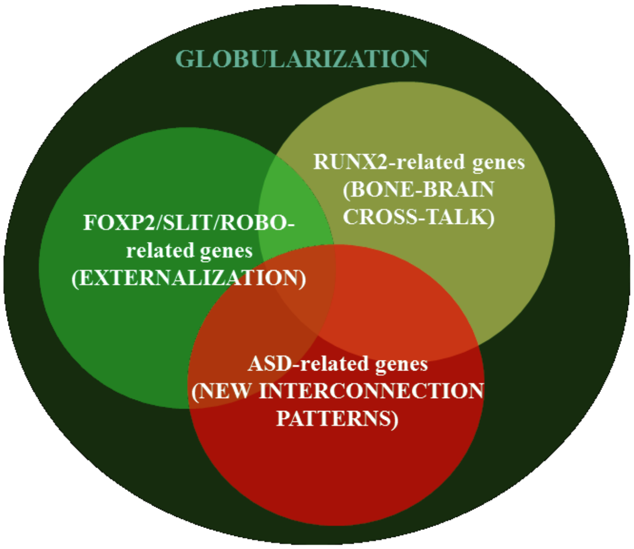

Figure S1. Three putative gene networks that may account for the emergence of language-readiness in our species. As noted in the main text, all of them include candidate genes for schizophrenia (based on Boeckx and Benítez-Burraco, 2014a,b and Benítez-Burraco and Boeckx, 2015a).

Regarding the genes clustered around RUNX2, we wish to note that *RUNX2* is listed among the genes associated with GAD1-dependent GABAergic dysfunction in schizophrenia (Benes et al. 2007). GAD1 regulatory network is important for the normal development of GABAergic neurons within the hippocampus (Pleasure et al. 2000, Ruzicka et al. 2015) and *GAD1* itself is a strong candidate for schizophrenia (Mitchell et al. 2015). Interestingly, some polymorphisms of *GAD1* affect to white matter organization and long-interval cortical inhibition in the dorsolateral prefrontal cortex of schizophrenia patients, and ultimately, to attentional processing and working memory (Lett et al. 2016). Other genes important for globularity and the emergence of our language-ready brain besides *RUNX2* interact with GAD1, including *FOXP2*, *DLX1*, and *DLX2*. Importantly, the promoter region of *RUNX2* shows strong signals of a selective sweep in AMHs (Green et al. 2010). Moreover, the interaction between RUNX2 and VDR (the 1α,25-dihydroxyvitamin D3 receptor) regulates the expression of both *SPAG5* and *SRGAP3* (Stephens and Morrison 2014). *SPAG5* has been selected in AMHs (Green et al. 2010) and encodes an interactor of the isoform B of USH2A (Kersten et al. 2012), the main candidate for Usher syndrome, a condition involving combined deaf-blindness and occasional schizophrenia-like symptoms (Domanico et al. 2012; see Leivada and Boeckx 2014 for detailed discussion). In turn, *SRGAP3* is related to both schizophrenia (Wilson et al. 2011; Waltereit et al. 2012) and severe mental retardation and absence of speech (Endris et al. 2002). Interestingly, one interactor of SRGAP3 during neuronal differentiation and neurite outgrowth, namely SRGAP2 (Ma et al. 2013), has been duplicated three times in humans (Sudmant et al. 2010). Both SRGAP2 and SRGAP3 interact with ROBO1 and affect the SLIT/ROBO pathway (Wong et al. 2001), important for the externalization of language, as noted above (see Boeckx and Benítez-Burraco 2014b for details). RUNX2 also interacts with *APOE* (Kuhlwilm et al. 2013), a gene related to encephalization and cognitive development in our clade (Bufill and Carbonell 2006) and part of the Reelin signalling cascade related to cognitive dysfunction in schizophrenia, including verbal memory deficits (Verbrugghe et al. 2012; Li et al. 2015). Interestingly, the allele ε4 of *APOE* (related to a higher risk for developing late onset Alzheimer's disease) has been shown to differentially affect high and low frequency bands in several areas of the brain, plausibly impacting negatively on the cognitive abilities of the carriers (Canuet et al. 2012, Cuesta et al. 2015a, Cuesta et al. 2015b, Prieto del Val et al. 2015). *NCAM1*, which encodes a protein involved in axonal and dendritic growth, synaptic plasticity, and cognition, is a potential target of RUNX2 too (Kuhlwilm et al. 2013), but also of FOXP2 (Konopka et al. 2009). *NCAM1* has been related to schizophrenia (Vawter et al. 2001; Atz et al. 2007) and working memory performance (Bisaz et al. 2013). Interestingly, it interacts with VCAM1, a protein that shows a fixed change (D414G) in AMHs compared to Neanderthals/Denisovans (Pääbo, 2014, Table S1). VCAM1 is involved in cell adhesion in the subventricular zone (Kokovay et al. 2012). In turn, *VCAM1* is upregulated by CLOCK (Gao et al. 2014), a circadian gene associated to schizophrenia (Zhang et al. 2011; Jung et al. 2014), and an interactor of RUNX2 (Reale et al. 2013). VCAN is also functionally linked to EGFR, another of RUNX2's targets (Kuhlwilm et al. 2013) and a candidate for schizophrenia too (Benzel et al. 2007), a link which reinforces the view that ERBB and NRG families are causative factors of the disease, as noted before. Among the genes belonging to the RUNX2 network we wish also highlight one finds *DLX1*, *DLX5*, and *DLX6*. Decreased expression of *DLX1* in the thalamus has been observed in schizophrenics (Kromkamp et al. 2003). Abnormal configuration of thalamic circuits is a hallmark of the disease, whereas changes in the thalamus are expected to have contributed to our mode of cognition (see Boeckx and Benítez-Burraco 2014a for details). DLX5 and DLX6 regulate GABAergic interneuron development (Cobos et al. 2006). Importantly, Dlx5/6(+/-) mice show and abnormal pattern of γ rhythms resulting from abnormalities in GABAergic interneurons, particularly fast-spiking interneurons, which impact on their cognitive flexibility (Cho et al. 2015). On the whole, the genes highlighted above are primarily related to the specification, migration and interconnection of

GABAergic neurons within the forebrain, to skull morphogenesis and to thalamic development, all of them aspects known to be impaired in schizophrenia. This circumstance reinforces the view that globularization was brought about by changes in genes that are involved in schizophrenia when mutated.

Regarding the network centered around FOXP2 and the ROBO/SLIT factors, we wish to mention that *FOXP2* has been recurrently associated to schizophrenia (Li et al. 2013) and to some of the changes observed in the brain of schizophrenics, including a reduction of grey matter in areas involved in language processing that may contributed to the verbal hallucinations that are a hallmark of the disease (Španiel et al. 2011). As noted above several targets of FOXP2 are related to schizophrenia (*CNTNAP2*, *DISC1*, *MEF2C*). Also some of its effectors are related to the disease. For example, sequence and copy number variations affecting *POU3F2* have been found in subjects with schizophrenia (Huang et al. 2005; Potkin et al. 2009). Importantly, the AMH POU3F2 is less efficient than the Neanderthal version in activating transcription of *FOXP2* (Maricic et al. 2013). POU3F2 regulates dopamine and serotonin synthesis (Nasu et al. 2014) and neuronal migration and identity in the neocortex (McEvilly et al. 2002; Sugitani et al. 2002). Likewise, FOXP2 regulates *MET* (Mukamel et al. 2011), a gene that influences schizophrenia risk and neurocognition (Burdick et al. 2010). Interestingly, *FOXP2* and some other candidates for schizophrenia reviewed above, like *CNTNAP2* and *DLX1*, are enriched ELAVL2 target genes (Konopka et al. 2012). *ELAVL2* encodes a splicing factor involved in cortical neurogenesis whose expression pattern has changed in humans (Konopka et al. 2012), and it is a candidate for schizophrenia too (Yamada et al. 2011). Likewise both *ROBO1* and *ROBO2*, core components of our network, have been proposed as schizophrenia-candidate genes (Benes et al. 2009, Potkin et al. 2009, 2010). Both genes are involved in thalamocortical axon development, which represent the major input to the neocortex, and modulate cognitive functions, consciousness and alertness (López-Bendito et al. 2007; Marcos-Mondéjar et al. 2012). Both genes are differentially expressed in areas important for singing in adult male zebra finches (Wang 2011). In humans, *ROBO1* has been associated with dyslexia and speech sound disorder (Hannula-Jouppi et al. 2005; Mascheretti et al. 2014), whereas *ROBO2* has been associated with expressive vocabulary growth in the normal population (St Pourcain et al. 2014), and linked to dyslexia (Fisher et al. 2002) and speech-sound disorder and reading (Stein et al. 2004).

Other ROBO/SLIT-related genes that belong to our network and that are also candidates for schizophrenia are *ABL1*, *AKT1*, *CTNNB1*, *DCC*, *EGR1*, *MAPK14*, and *PCM1*. *ABL1* is involved in cell differentiation, division, and adhesion important for the regulation and/or the activation of auditory networks within the thalamus (Habib et al. 2013) and is differentially expressed in the hippocampus of schizophrenics (Benes et al. 2009). *AKT1* is involved in neuronal survival and bone formation (Dudek et al. 1997; Peng et al. 2003). In humans mutations in *AKT1* have been associated to schizophrenia (Emamian et al. 2004) and Proteus syndrome (Cohen 2014). Interestingly, reduced *Akt1* expression in mutant *Akt1*(+/-) and *Akt1*(-/-) results in increased reduction in gamma synchrony and theta suppression following ketamine administration (Featherstone et al. 2013). *CTNNB1*¸ related to schizophrenia (like other components of the Wnt/β-catenin pathway) (Levchenko et al. 2015), interacts with *PCDH11X/Y*, the gene pair that has undergone accelerated evolution in our lineage (Williams et al. 2006) and that has been linked to language acquisition delay (Speevak and Farrell 2011) and to schizophrenia and language evolution, as noted above (see Crow 2013 for discussion). *DCC* is involved in thalamocortical axon projections and the organization of dopaminergic circuits within the cortex (Braisted et al. 2000; Grant et al. 2007). DCC contributes to the genetic basis behind individual differences in susceptibility to schizophrenia (Grant et al. 2007; Grant et al. 2012). Importantly, an hCONDEL (shared with Neanderthals) exist in a region upstream of *DCC* (McLean et al. 2011). *EGR1* is found differentially expressed in the prefrontal cortex of schizophrenics (Pérez-Santiago et al. 2012). This gene encodes a

transcription factor involved in neuronal plasticity and memory consolidation (Veyrac et al. 2014). EGR1 downregulates *PLAUR* (Matsunoshita et al. 2011), a target of FOXP2 (Roll *et al.* 2010) which encodes an effector of SRPX2, another of FOXP2 targets (Royer-Zemmour et al. 2008) and a candidate for rolandic epilepsy and speech dyspraxia (Roll et al. 2006). *MAPK14* encodes an interactor of both ABL1 and AKT1 involved in cellular proliferation and differentiation, and it is also a candidate for brain changes in schizophrenia (Onwuameze et al. 2013). Mapk14 signaling pathway has been related to kainite-induced epilepsy in mice (Namiki et al. 2007). Finally, *PCM1*, which encodes a centrosome protein that interacts with SLIT1 and that is necessary for neuronal migration, shows a differential expression in mammalian vocal learners (Wang 2011). PCM1 also interacts with DISC1 in the centrosome, mimicking its effects on neural migration and cortical development (Kamiya et al. 2008). On the whole, these genes are prominent signatures of vocal learning, important for the externalization component of the language-ready brain, which is also impaired in schizophrenia, as described in sections 2 and 3.

Regarding the genes clustered around *AUTS2*, we wish to highlight that AUTS2 itself, though a strong candidate for autism, has been recently associated with the disease (Zhang et al. 2014). The first half of *AUTS2* displays the strongest signal of positive selection in AMHs compared to Neanderthals and contains several human accelerated regions which include enhancers that seem to be active in the brain (Green et al. 2010; Oksenberg et al. 2013). AUTS2 interacts with many proteins involved in brain development and function that are encoded by candidate genes for several neurodevelopmental disorders affecting cognition and language (reviewed by Oksenberg and Ahituv 2013), including RELN and TBR1. TBR1 is a partner of DYRK1A, encoded by a gene that contains a region showing signals of strong selection in AMHs (Green et al. 2010) and whose mutations affect speech abilities (Van Bon et al. 2011; Courcet et al. 2012). DYRK1A regulates *GAD1* (Souchet et al. 2014) and it is important for the control of balance between excitation and inhibition in the brain and for synaptogenesis and synaptic plasticity (and, ultimately, for learning and memory) (Hämmerle et al. 2003; Souchet et al. 2014). Moreover, DYRK1A directly phosphorylates SIRT1 and also promotes deacetylation of TP53. Both *SIRT1* and *TP53* are candidates for schizophrenia (Ni et al. 2005; Kishi et al. 2011; Wang et al. 2015). Interestingly, SIRT1 is an effector of several genes under selection in modern populations that show non-fixed changes in their coding regions compared to Neanderthals and Denisovans, like *BAZ2A* and *NR1H2* (Prüfer et al. 2014). Interestingly too, it has been recently shown that the down-regulation of *SIRT1* (via miR-199a-5p) induces seizures and seizure damage in rats (Wang et al. 2016). *SIRT1* is functionally related to *MEF2A* too (Gracia-Sancho et al., 2010), an important gene implicated in differences between human and chimpanzee prefrontal cortex development (Liu et al. 2012) and that shows signals of recent positive selection (Somel et al. 2013). According to Liu and colleagues (2012), these differences may account for the presumed faster cortical synaptic development in Neanderthals. Notably, a binding site for MEF2A has been linked to formal thought disorder (Thygesen et al. 2015). Likewise, TP53 exhibits a non-fixed change (P72R) compared to Neanderthals/Denisovans (Paskulin et al. 2012) and the expression pattern of the human gene differs from the patterns observed in other primates (Konopka et al. 2012). Risk alleles for TP53 seem to contribute to the reduced metabolic activity and the reduced white matter volumes observed in the frontal lobe of schizophrenics (Molina et al. 2011). On the whole, these changes seemingly contributed to the refinement of the changes that brought about modern cognition and enhanced speech abilities in humans.

Some other genes relevant for brain function that show changes that occurred after the split between AMHs and Neanderthals/Denisovans, and that reinforce the links between the three sets of genes

highlighted above, are candidates for schizophrenia. We will focus only on those belonging to the CDC42 signaling pathway and the SHH-GLI signaling pathway. Firstly, *CDC42* is required for proper cortical interneuron migration (Katayama et al. 2013). Some risk polymorphisms for schizophrenia reduce the expression of *CDC42* (Gilks et al. 2012). Specifically, the downregulation of the gene in the dorsolateral prefrontal cortex appears to contribute to the reduction of dendritic spines on pyramidal cells and, ultimately, to the cognitive dysfunction characteristic of the disease (Datta et al. 2015). For our purposes, it is useful to note that altered expression of the gene in the hippocampus may be caused by the downregulation of some micro-RNAS, particularly of miR-185, found in the critical region deleted in 22q11.2 deletion syndrome (Forstner et al. 2013). Another target of miR-185 is *RHOA*, also altered in schizophrenia and involved in cortical interneuron migration, and is one of the genes showing strong signals of positive selection in AMHs compared to Neanderthals (Green et al. 2010). Two members of the CDC42 signaling pathway are also altered in schizophrenia: *CDC42EP4* (Datta et al. 2015), which is hypermethylated in AMHs compared to Denisovans (Gokhman et al. 2014), and *CDC42BPB* (Narayan et al. 2008), which is a target of FOXP2 (Spiteri et al. 2007). *ARHGAP32* is another partner of CDC42 related to schizophrenia and schizotypal personality traits (Ohi et al. 2012). It encodes a receptor of NMDA that modulates Rho-GTPase activity and it bears a fixed change (E1489D) in AMHs compared to Denisovans (Meyer et al. 2012). These data suggest that synergistic alterations in CDC42 signaling pathway may contribute to spine deficits in cells in schizophrenia and that this pathway has changed in our species. Concerning the SHH-GLI pathway, we expect it to have played a key role in the anatomical and physiological events leading to globularization (see Boeckx et al. submitted), but it also contributes to the pathobiology of schizophrenia (Boyd et al. 2015). SHH upregulates *DISC1* (Boyd et al. 2015). DISP1, one component of the SHH signalling network, shows a fixed change in AMHs (Green et al. 2010). SOX factors provide positional information in SHH-directed neural patterning together with GLI factors and some of them are related to schizophrenia. Hence, SOX10 is found to be hypermethylated in the brain of schizophrenics (Iwamoto et al. 2005, Wockner et al. 2014). Together with DISC1 it acts as negative regulator of oligodendrocyte differentiation (Drerup et al. 2009, Hattori et al. 2014). SOX2 is also involved in the enhancer effect of human endogenous retroviruses (HERVs) on brain genes related to schizophrenia, specifically on *PRODH* (Suntsova et al. 2013). Schizophrenia has been claimed to result in part from epigenetic changes that deregulate HERV-activity (Frank et al. 2005; Diem et al. 2012). HERVs are non-coding DNA remnants of retroviral infections occurred during primate evolution and seem to have fueled genomic rearrangements associated with or subsequent to speciation events (Böhne et al. 2008), so we expect them to have contributed as well to language evolution (see Benítez-Burraco and Uriagereka 2016 for discussion). Interestingly, a recent study by Castro-Nallar (2015) also found intriguing evidence of diversity in the schizophrenic oropharyngeal microbiome, with *Ascomycota* being more dominant and lactic acid being more abundant in schizophrenics than controls. The differences in bacteria between the two groups was clear, although its functional significance remains obscure. The microbiome has been shown to influence human cognition and behaviour through imbalances in the microbiota-gut-central nervous system axis (Foster and McVey Neufeld 2013, Hsiao et al. 2013). Other causal relations between schizophrenia and the 'phageome' have been posited (Yolken et al. 2015), and a number of studies connecting immune disorders and schizophrenia have also been forthcoming (reviewed by Severance et al. 2013). The behavioural and cognitive alterations seen in the microbiome can be changed via probiotic and antibiotic interventions (Jakobsson et al. 2010), and so an understanding of the relationship between cognition and viral, bacterial and fungal profiles could lead to successful remedial action. Together with Benítez-Burraco and Uriagereka's (2016) claim that brain/immune system crosstalk led to alterations in brain connectivity giving rise to language, the microbiome appears to be a potentially fruitful area of research into the neurocognitive origins of schizophrenia.

B. *Candidates genes for schizophrenia selected in modern humans (according to Srinivasan et al. 2015) that are involved in language evolution (according to Benítez-Burraco and Boeckx)*

As noted in the main text, many candidates for schizophrenia that show signals of positive selection in modern humans compared to Neanderthals are also candidates for language impairment and/or are functionally related to the set of genes involved in the evolution of language-readiness. We have highlighted several of them as new candidates for language dysfunction in schizophrenia, including *FOXP1*, *GATAD2B*, *MEF2C*, *NRG3*, and *NRXN1*.

- *FOXP1* encodes an interactor of FOXP2 (Li et al. 2004). *FOXP1* is expressed in areas relevant to cortico-laryngeal connections (Inoue et al. 2008) and its mutations cause language impairment, intellectual disability, and autism (Hamdan et al. 2010; Sollis et al. 2016). *FOXP1* is mentioned among the top five percent regions showing signals of positive selection in AMHs (Green et al. 2010).
- *GATAD2B* encodes a zinc protein involved in chromatin modification and regulation of gene expression; mutations in *GATAD2B* impact synaptic growth and function (Willemsen et al. 2013) and have been related to mental retardation, intellectual disability, and learning problems (De Ligt et al. 2012; Hamdan et al. 2014; Roberts et al. 2014), and specifically, to limited speech (Willemsen et al. 2013).
- *MEF2C* encodes a trans-activating and DNA binding protein involved in early neurogenesis, neuronal migration, and differentiation. Mutations in *MEF2C* cause absent speech, severe mental retardation, and epilepsy (Bienvenu et al. 2013). *MEF2C* is a target of FOXP2 in the basal ganglia (Spiteri et al. 2007).
- *NRG3* is a promising candidate for atypical neurodevelopmental outcomes (including cognitive anomalies and abnormal infant behaviour) that may affect preterm infants in absence of rare genetic diseases (Blair et al. 2016). Deletions and duplications involving *NRG3* give rise to speech delay (van Bon et al. 2011). In conjunction with NRG1 and their receptor ERBB4 (reviewed below), NRG3 regulates the migration of GABAergic interneurons from ganglionic eminences to their cortical targets (Li et al. 2012).
- *NRXN1* encodes one of the largest known neurexins, a presynaptic cell adhesion molecule important for synaptic activity, neuritogenesis, and neuronal network assembly related to neocortical development (Südhof, 2008; Gjørlund et al. 2012; Jenkins et al. 2015). Mutations in *NRXN1* impact speech severely, although give rise to mild motor delay only (Zweier 2012).

C. *Functional interactions predicted by String 10 among candidates for the evolution of language, candidate genes for schizophrenia, and genes important for brain rhythmicity.*

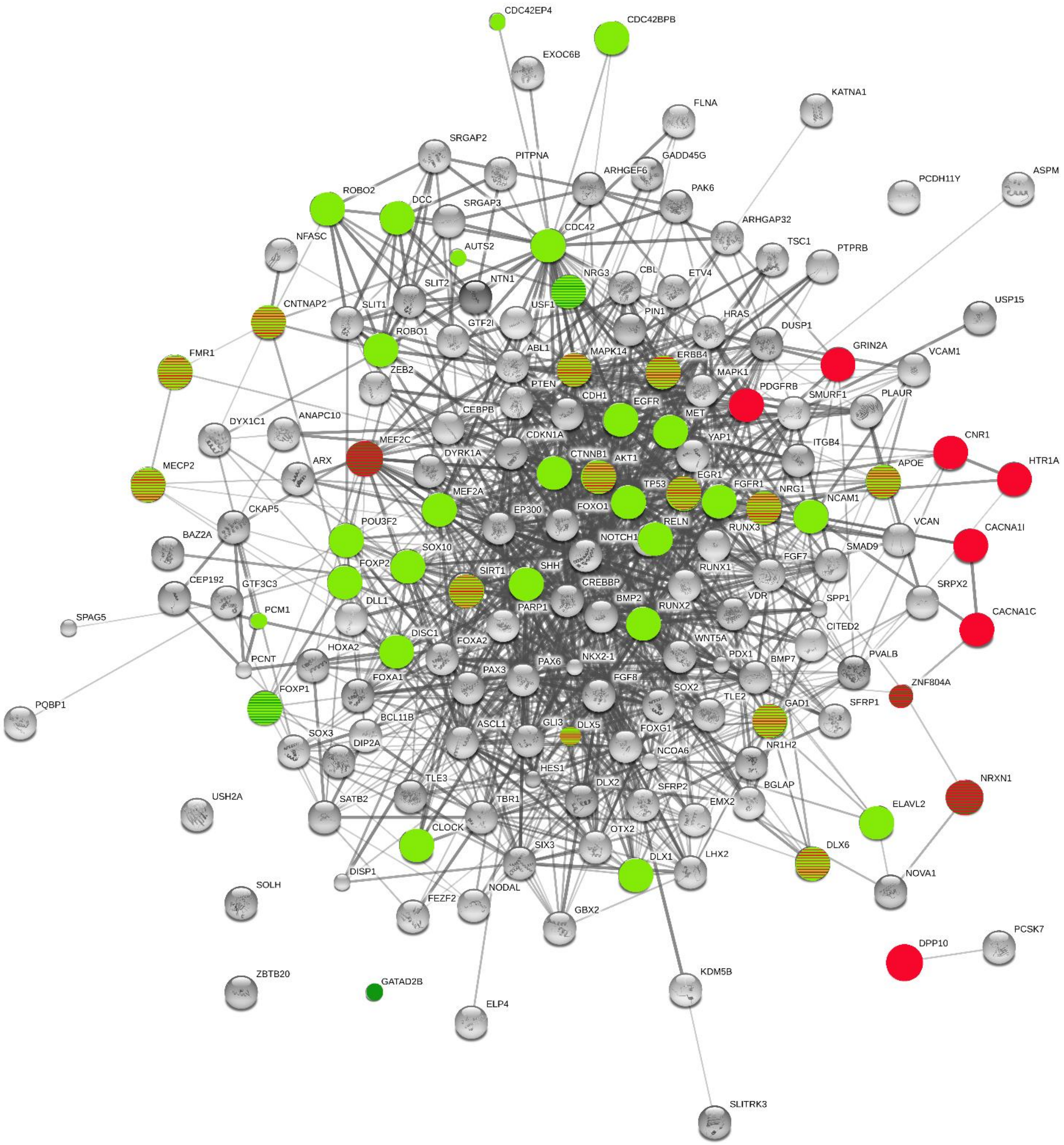

Figure 2. Functional links predicted by String 10 among candidates for the evolution of language, candidate genes for schizophrenia, and genes important for brain rhythmicity.

This figure shows the functional links among candidates for the evolution of language, candidate genes for schizophrenia, and genes important for brain rhythmicity, as predicted by String 10 (http://string-db.org/). Candidates have been colored according to the following criteria:

- Candidate genes for the evolution of language (as posited by Boeckx and Benítez-Burraco 2014a,b, and Benítez-Burraco and Boeckx 2015) that are also candidates for schizophrenia are colored in light green. All these genes are displayed in table 2 and have been described in section A above, with the exception of *EGR1*, *FGFR1*, *FMR1*, *MECP2*, and *TGF* (these genes, particularly *FMR1* and *MECP2*, have been associated to well-known cognitive diseases and to anomalies in the normal pattern of brain activity).
- Genes related to brain rhythms are colored in red, but they appear stripped in red and light green if they belong to any of the interactomes important for language evolution.
- Candidate genes for schizophrenia showing signals of positive selection in AMHs according to Srinivasan et al. (2015) are colored in dark green, but they appear stripped in dark and light green if they also belong to the list of candidates for language evolution.
- Three genes, namely, *MEF2C*, *NRXN1*, and *ZNF804A*, are stripped in red and dark green, meaning that they are both related to brain oscillations and have been selected in AMHs, although we have not considered it yet as part of the putative interactome for the language-ready brain.
- Candidate genes for the evolution of language that are not candidates for schizophrenia, nor are involved in brain oscillations are shadowed in gray.

Stronger associations between proteins are represented by thicker lines. The medium confidence value was .0400. This means that one should expect a 40% probability that a predicted link exists between two enzymes in the same metabolic map in the KEGG database (http://www.genome.jp/kegg/pathway.html). This lower value enables to find a greater number of potential interactions among proteins, although it is then compensated for by checking whether the predicted interactions can be confirmed in the literature or in databases provided by functional assays.

We wish note that String 10 predicts associations between proteins that derive from a limited set of databases: genomic context, high-throughput experiments, conserved coexpression, and the knowledge previously gained from text mining (Szklarczyk et al. 2015). This is why the figure does not represent a fully connected graph (evidence for additional links are provided in the supplementary materials). Importantly, the diagram only represents the potential connectivity between the involved proteins, which has to be mapped onto particular biochemical networks, signaling pathways, cellular properties, aspects of neuronal function, or cell-types of interest that can be confidently related to aspects of language development and function. Nonetheless, we wish note that the 6-top GO biological processes in which core candidates genes for language evolution are predicted to be involved (according to Panther [http://pantherdb.org]) are: multicellular organismal process (GO:0032501), response to stimulus (GO:0050896), immune system process (GO:0002376), apoptotic process (GO:0006915), cellular component organization or biogenesis (GO:0071840), and biological adhesion (GO:0022610). Likewise, the 6 most significant GO pathways in which core candidates for language evolution are predicted to be involved (according to Panther) are: gonadotropin releasing hormone receptor pathway (P06664), TGF-beta signaling pathway (P00052), angiogenesis (P00005), Wnt signaling pathway (P00057), EGF receptor

signaling pathway (P00018), and axon guidance mediated by Slit/Robo (P00008) (see section A above for some additional concerns regarding schizophrenia).

References

Atz, M. E., Rollins, B., and Vawter, M. P. (2007). NCAM1 association study of bipolar disorder and schizophrenia: polymorphisms and alternatively spliced isoforms lead to similarities and differences. *Psychiatr. Genet.* 17, 55-67.

Benes, F. M., Lim, B., and Subburaju, S. (2009). Site-specific regulation of cell cycle and DNA repair in post-mitotic GABA cells in schizophrenic versus bipolars. *Proc. Natl. Acad. Sci. U. S. A.* 106, 11731-6.

Benes, F. M., Lim, B., Matzilevich, D., Walsh, J. P., Subburaju, S., and Minns, M. (2007). Regulation of the GABA cell phenotype in hippocampus of schizophrenics and bipolars. *Proc. Natl. Acad. Sci. U.S.A.* 104, 10164-9.

Benítez-Burraco, A. and Boeckx, C. (2015). Possible functional links among brain- and skull-related genes selected in modern humans. *Front. Psychol.* 6, 794.

Benítez-Burraco, A., and Uriagereka, J. (2016). The immune syntax revisited: opening new windows on language evolution. *Front. Mol. Neurosci.* 8, 84.

Benzel, I., Bansal, A., Browning, B. L., Galwey, N. W., Maycox, P. R., McGinnis, R., et al. (2007). Interactions among genes in the ErbB-Neuregulin signalling network are associated with increased susceptibility to schizophrenia. *Behav. Brain Funct.* 3, 31.

Bienvenu, T., Diebold, B., Chelly, J., and Isidor, B. (2013). Refining the phenotype associated with MEF2C point mutations. *Neurogenetics* 14, 71-5.

Bisaz, R., Boadas-Vaello, P., Genoux, D., and Sandi C. (2013). Age-related cognitive impairments in mice with a conditional ablation of the neural cell adhesion molecule. *Learn. Mem.* 20, 183-93.

Blair, L. M., Pickler, R. H., and Anderson, C. (2016). Integrative review of genetic factors influencing neurodevelopmental outcomes in preterm infants. *Biol. Res. Nurs*. 18, 127-37.

Boeckx, C. and Benítez-Burraco, A. (2014a). The shape of the human language-ready brain. *Front. Psychol.* 5, 282.

Boeckx, C. and Benítez-Burraco, A. (2014b). Globularity and language-readiness: Generating new predictions by expanding the set of genes of interest. *Front. Psychol.* 5, 1324.

Boeckx, C., Theofanopoulou, C., and Benítez-Burraco, A. (submitted). GLI transcription factors and globularization of the human brain.

Böhne, A., Brunet, F., Galiana-Arnoux, D., Schultheis, C., and Volff, J. N. (2008). Transposable elements as drivers of genomic and biological diversity in vertebrates. *Chromosome Res.* 16, 203-15.

Booth, C. A., Brown, J. T., and Randall, A. D. (2014). Neurophysiological modification of CA1 pyramidal neurons in a transgenic mouse expressing a truncated form of disrupted-in-schizophrenia 1. *Eur. J. Neurosci.* 39, 1074-90.

Boyd, P. J., Cunliffe, V. T., Roy, S., and Wood, J. D. (2015). Sonic hedgehog functions upstream of disrupted-in-schizophrenia 1 (disc1): implications for mental illness. *Biol. Open* 4, 1336-43.

Braisted, J.E., Ringstedt, T., and O'Leary, D.D.M. (2009). Slits are chemorepel- lents endogenous to hypothalamus and steer thalamocortical axons into ventral telencephalon. *Cereb. Cortex* 19, i144–i151.

Bufill, E., and Carbonell, E. (2006). Apolipoprotein E polymorphism and neuronal plasticity. *Am. J. Hum. Biol.* 18, 556-558.

Burdick, K. E., DeRosse, P., Kane, J. M., Lencz, T., and Malhotra, A.K. (2010). Association of genetic variation in the MET proto-oncogene with schizophrenia and general cognitive ability. *Am. J. Psychiatry* 167, 436-43.

Canuet, L., Tellado, I., Couceiro, V., Fraile, C., Fernandez-Novoa, L., Ishii, R. et al. (2012). Resting-state network disruption and APOE genotype in Alzheimer's disease: a lagged functional connectivity study. *PLoS One* 7, e46289.

Castro-Nallar, E., Bendall, M.L., Pérez-Losada, M., Sabuncyan, S., Severance, E.G., Dickerson, F.B., et al. (2015) Composition, taxonomy and functional diversity of the oropharynx microbiome in individuals with schizophrenia and controls. *PeerJ* 3, e1140.

Cho, K. K., Hoch, R., Lee, A. T., Patel, T., Rubenstein, J. L, and Sohal, V. S. (2015). Gamma rhythms link prefrontal interneuron dysfunction with cognitive inflexibility in Dlx5/6(+/-) mice. *Neuron* 85, 1332-43.

Cobos, I., Long, J. E., Thwin, M. T., and Rubenstein, J. L. (2006). Cellular patterns of transcription factor expression in developing cortical interneurons. Cereb. Cortex. *16*, i82-88.

Courcet, J. B., Faivre, L., Malzac, P., Masurel-Paulet, A., López, E., Callier, P., et al. (2012). The DYRK1A gene is a cause of syndromic intellectual disability with severe microcephaly and epilepsy. *J. Med. Genet.* 49, 731-6.

Cuesta, P., Barabash, A., Aurtenetxe, S., Garcés, P., López, M. E., Bajo, R. et al. (2015b). Source analysis of spontaneous magnetoencephalograpic activity in healthy aging and mild cognitive impairment: influence of apolipoprotein E polymorphism. *J. Alzheimers Dis.* 43, 259-73.

Cuesta, P., Garcés, P., Castellanos, N. P., López, M. E., Aurtenetxe, S., Bajo, R. et al. (2015a). Influence of the APOE ε4 allele and mild cognitive impairment diagnosis in the disruption of the MEG resting state functional connectivity in sources space. *J. Alzheimers Dis.* 44, 493-505.

Datta, D., Arion, D., Corradi, J. P., and Lewis, D. A. (2015). Altered expression of CDC42 signaling pathway components in cortical layer 3 pyramidal cells in schizophrenia. *Biol. Psychiatry* 78, 775-85.

De Ligt, J., Willemsen, M. H., van Bon, B. W. M., Kleefstra, T., Yntema, H. G., Kroes T, et al. (2012). Diagnostic exome sequencing in persons with severe intellectual disability. *New Engl. J. Med.* 367, 1921–1929.

Diem, O., Schäffner, M., Seifarth, W., and Leib-Mösch, C. (2012). Influence of antipsychotic drugs on human endogenous retrovirus (HERV) transcription in brain cells. *PLoS One* 7, e30054.

Domanico, D., Fragiotta, S., Trabucco, P., Nebbioso, M., and Vingolo, E. M. (2012). Genetic analysis for two italian siblings with usher syndrome and schizophrenia. *Case Rep. Ophthalmol. Med.* 2012, 380863.

Drerup, C. M., Wiora, H. M., Topczewski, J., and Morris, J. A. (2009). Disc1 regulates foxd3 and sox10 expression, affecting neural crest migration and differentiation. *Development* 136, 2623-32.

Dudek, H., Datta, S. R., Franke, T. F., Birnbaum, M. J., Yao, R., Cooper, G. M., et al. (1997). Regulation of neuronal survival by the serine-threonine protein kinase Akt. *Science* 275, 661-5.

Emamian, E. S., Karayiorgou, M., and Gogos, J. A. (2004). Decreased phosphorylation of NMDA receptor type 1 at serine 897 in brains of patients with schizophrenia. *J. Neurosci.* 24, 1561-4.

Endris, V., Wogatzky, B., Leimer, U., Bartsch, D., Zatyka, M., Latif, F., et al. (2002). The novel Rho-GTPase activating gene MEGAP/ srGAP3 has a putative role in severe mental retardation. *Proc. Natl. Acad. Sci. U.S.A.* 99, 11754–9.

Featherstone, R. E., M Tatard-Leitman, V., Suh, J. D., Lin, R., Lucki, I., and Siegel, S. J. (2013). Electrophysiological and behavioral responses to ketamine in mice with reduced Akt1 expression. *Psychopharmacology* 227, 639-49

Fisher S. E., Francks, C., Marlow, A. J., MacPhie, I. L., Newbury, D. F., Cardon, L. R., et al. (2002). Independent genome-wide scans identify a chromosome 18 quantitative-trait locus influencing dyslexia. *Nat. Genet.* 30, 86–91.

Forstner, A. J., Degenhardt, F., Schratt, G., and Nöthen, M. M. (2013). MicroRNAs as the cause of schizophrenia in 22q11.2 deletion carriers, and possible implications for idiopathic disease: a mini-review. *Front. Mol. Neurosci.* 6, 47.

Frank, O., Giehl, M., Zheng, C., Hehlmann, R., Leib-Mösch, C., and Seifarth, W. (2005). Human endogenous retrovirus expression profiles in samples from brains of patients with schizophrenia and bipolar disorders. *J. Virol.*79, 10890-901.

Gao, Y., Meng, D., Sun, N., Zhu, Z., Zhao, R., Lu, C., et al. (2014). Clock upregulates intercellular adhesion molecule-1 expression and promotes mononuclear cells adhesion to endothelial cells. *Biochem. Biophys. Res. Commun.* 443, 586-91.

Gilks, W. P., Hill, M., Gill, M., Donohoe, G., Corvin, A. P., and Morris, D. W. (2012). Functional investigation of a schizophrenia GWAS signal at the CDC42 gene. *World J. Biol. Psychiatry* 13, 550-4.

Gjørlund, M. D., Nielsen, J., Pankratova, S., Li, S., Korshunova, I., Bock, E., et al. (2012). Neuroligin-1 induces neurite outgrowth through interaction with neurexin-1β and activation of fibroblast growth factor receptor-1. *FASEB J.* 26, 4174-86.

Gokhman, D., Lavi, E., Prüfer, K., Fraga, M. F., Riancho, J, A., Kelso, J., *et al.* (2014). Reconstructing the DNA methylation maps of the Neandertal and the Denisovan. *Science* 344, 523-527.

Gracia-Sancho, J., Villarreal, G. Jr., Zhang, Y., and García-Cardeña, G. (2010). Activation of SIRT1 by resveratrol induces KLF2 expression conferring an endothelial vasoprotective phenotype. *Cardiovasc. Res.* 85, 514-519.

Grant, A., Fathalli, F., Rouleau, G., Joober, R., and Flores, C. (2012). Association between schizophrenia and genetic variation in DCC: a case-control study. *Schizophr. Res.* 137, 26-31.

Grant, A., Hoops, D., Labelle-Dumais, C., Prévost, M., Rajabi, H., Kolb, B., et al. (2007). Netrin-1 receptor-deficient mice show enhanced mesocortical dopamine transmission and blunted behavioural responses to amphetamine. *Eur. J. Neurosci.* 26, 3215-28.

Green, R. E., Krause, J., Briggs, A. W., Maricic, T., Stenzel, U., Kircher, M., et al. (2010). A draft sequence of the Neandertal genome. *Science* 328, 710-22.

Habib, M. R., Ganea, D. A., Katz, I. K., and Lamprecht, R. (2013). ABL1 in thalamus is associated with safety but not fear learning. *Front. Syst. Neurosci.* 7, 5.

Hamdan, F. F., Daoud, H., Rochefort, D., Piton, A., Gauthier, J., Langlois, M., et al. (2010). De novo mutations in FOXP1 in cases with intellectual disability, autism, and language impairment. *Am. J. Hum. Genet.* 87, 671-678.

Hamdan, F. F., Srour, M., Capo-Chichi, J. M., Daoud, H., Nassif, C., Patry, L., et al. (2014). De novo mutations in moderate or severe intellectual disability. *PLoS Genet.* 10, e1004772.

Hämmerle, B., Carnicero, A., Elizalde, C., Ceron, J., Martínez, S., and Tejedor, F. J. (2003). Expression patterns and subcellular localization of the Down syndrome candidate protein MNB/DYRK1A suggest a role in late neuronal differentiation. *Eur. J. Neurosci.* 17, 2277-86.

Hannula-Jouppi, K., Kaminen-Ahola, N., Taipale, M., Eklund, R., Nopola-Hemmi, J., Kääriäinen, H., *et al.* (2005). The axon guidance receptor gene ROBO1 is a candidate gene for developmental dyslexia. *PLoS Genet.* 1, e50.

Hattori, T., Shimizu, S., Koyama, Y., Emoto, H., Matsumoto, Y., Kumamoto, N. et al. (2014). DISC1 (disrupted-in-schizophrenia-1) regulates differentiation of oligodendrocytes. *PLoS One* 9, e88506.

Huang, Y. T., Iwamoto, K., Kurosaki, T., Nasu, M., and Ueda, S. (2005). The neuronal POU transcription factor Brn-2 interacts with Jab1, a gene involved in the onset of neurodegenerative diseases. *Neurosci. Lett.* 382, 175-8.

Inoue, K. I., Shiga, T., and Ito, Y. (2008). Runx transcription factors in neuronal development. *Neural Dev.* 3, 8104-3.

Iwamoto, K., Bundo, M., Yamada, K., Takao, H., Iwayama-Shigeno, Y., Yoshikawa, T., et al. (2005). DNA methylation status of SOX10 correlates with its downregulation and oligodendrocyte dysfunction in schizophrenia. *J. Neurosci.* 25, 5376-81.

Jakobsson, H.E., Jernberg, C., Andersson, A.F., Sjölund-Karlsson, M., Jansson, J.K., and Engstrand, L. (2010). Short-term antibiotic treatment has differing long-term impacts on the human throat and gut microbiome. *PLoS ONE* 5, e9836.

Jenkins, A. K., Paterson, C., Wang, Y., Hyde, T. M., Kleinman, J. E., and Law, A. J. (2015). Neurexin 1 (NRXN1) splice isoform expression during human neocortical development and aging. *Mol. Psychiatry* doi: 10.1038/mp.2015.107.

Jung, J. S., Lee, H. J., Cho, C. H., Kang, S. G., Yoon, H. K., Park, Y. M., et al. (2014). Association between restless legs syndrome and CLOCK and NPAS2 gene polymorphisms in schizophrenia. *Chronobiol. Int.* 31, 838-44.

Kamiya, A., Tan, P. L., Kubo, K., Engelhard, C., Ishizuka, K., Kubo, A., et al. (2008). Recruitment of PCM1 to the centrosome by the cooperative action of DISC1 and BBS4: a candidate for psychiatric illnesses. *Arch. Gen. Psychiatry* 65, 996-1006.

Katayama, K., Imai, F., Campbell, K., Lang, R. A., Zheng, Y., and Yoshida Y. (2013). RhoA and Cdc42 are required in pre-migratory progenitors of the medial ganglionic eminence ventricular zone for proper cortical interneuron migration. *Development.* 140, 3139-45.

Kersten, F. F., van Wijk, E., Hetterschijt, L., Bauβ, K., Peters, T. A., Aslanyan, M. G., et al. (2012). The mitotic spindle protein SPAG5/Astrin connects to the Usher protein network postmitotically. *Cilia* 1, 2.

Kishi, T., Fukuo, Y., Kitajima, T., Okochi, T., Yamanouchi, Y., Kinoshita, Y., et al. (2011). SIRT1 gene, schizophrenia and bipolar disorder in the Japanese population: an association study. *Genes Brain Behav.* 10, 257-63.

Kokovay, E., Wang, Y., Kusek, G., Wurster, R., Lederman, P., Lowry, N., et al. (2012). VCAM1 is essential to maintain the structure of the SVZ niche and acts as an environmental sensor to regulate SVZ lineage progression. *Cell Stem Cell.* 11, 220-30.

Konopka, G., Bomar, J. M., Winden, K., Coppola, G., Jonsson, Z. O., Gao, F., et al. (2009). Human-specific transcriptional regulation of CNS development genes by FOXP2. *Nature* 462, 213-17.

Konopka, G., Friedrich, T., Davis-Turak, J., Winden, K., Oldham, M. C., Gao, F.,et al. (2012). Human-specific transcriptional networks in the brain. *Neuron* 75, 601-17.

Kromkamp, M., Uylings, H. B., Smidt, M. P., Hellemons, A. J., Burbach, J. P., and Kahn, R. S. (2003). Decreased thalamic expression of the homeobox gene DLX1 in psychosis. *Arch. Gen. Psychiatry* 60, 869-74.

Kuhlwilm, M., Davierwala, A., and Pääbo, S. (2013). Identification of putative target genes of the transcription factor RUNX2. *PLoS One* 8, e83218.

Leivada, E. and Boeckx, C. (2014). Schizophrenia and cortical blindness: Protective effects and implications for language. *Front. Hum. Neurosci.* 8, 940.

Lett, T. A., Kennedy, J. L., Radhu, N., Dominguez, L. G., Chakravarty, M. M., Nazeri, A., et al. (2016). Prefrontal white matter structure mediates the influence of GAD1 on working memory. *Neuropsychopharmacology* doi: 10.1038/npp.2016.14.

Levchenko, A., Davtian, S., Freylichman, O., and Zagrivnaya, M., Kostareva, A., and Malashichev, Y. (2015). Beta-catenin in schizophrenia: Possibly deleterious novel mutation. *Psychiatry Res.* 228, 843-8.

Li, H., Chou, S. J., Hamasaki, T., Perez-Garcia, C. G. and O'Leary, D. D. (2012). Neuregulin repellent signaling via ErbB4 restricts GABAergic interneurons to migratory paths from ganglionic eminence to cortical destinations. *Neural Dev.* 7, 10.

Li, S., Weidenfeld, J., and Morrisey, E. E. (2004). Transcriptional and DNA binding activity of the Foxp1/2/4 family is modulated by heterotypic and homotypic protein interactions. *Mol. Cell. Biol.* 24, 809-822.

Li, T., Zeng, Z., Zhao, Q., Wang, T., Huang, K., Li, J., et al. (2013). FoxP2 is significantly associated with schizophrenia and major depression in the Chinese Han population. *World J. Biol. Psychiatry* 14, 146-50.

Li, W., Guo, X., and Xiao, S. (2015). Evaluating the relationship between reelin gene variants (rs7341475 and rs262355) and schizophrenia: A meta-analysis. *Neurosci. Lett.* 609, 42-7.

Liu, X., Somel, M., Tang, L., Yan, Z., Jiang, X., Guo, S. et al. (2012). Extension of cortical synaptic development distinguishes humans from chimpanzees and macaques. *Genome Res.*22, 611-22.

López-Bendito, G., Flames, N., Ma, L., Fouquet, C., Di Meglio, T., Chedotal, A., et al. (2007). Robo1 and Robo2 cooperate to control the guidance of major axonal tracts in the mammalian forebrain. *J. Neurosci.* 27, 3395-407.

Ma, Y., Mi, Y.-J., Dai, Y.-K., Fu, H.-L., Cui, D.-X. and Jin,, W. L. (2013). The inverse F-BAR domain protein srGAP2 acts through srGAP3 to modulate neuronal differentiation and neurite outgrowth of mouse neuroblastoma cells. PLoS One 8, e57865.

Marcos-Mondéjar, P., Peregrín, S., Li, J. Y., Carlsson, L., Tole, S., and López-Bendito G. (2012). The lhx2 transcription factor controls thalamocortical axonal guidance by specific regulation of robo1 and robo2 receptors. *J. Neurosci.* 32, 4372-85.

Maricic, T., Günther, V., Georgiev, O., Gehre, S., Curlin, M., Schreiweis, C., *et al.* (2013). A recent evolutionary change affects a regulatory element in the human FOXP2 gene. *Mol. Biol. Evol.* 30, 844-852.

Mascheretti, S., Riva, V., Giorda, R., Beri, S., Lanzoni, L. F., Cellino, M. R., et al. (2014). KIAA0319 and ROBO1: evidence on association with reading and pleiotropic effects on language and mathematics abilities in developmental dyslexia. *J. Hum. Genet.* 59, 189-97.

Matsunoshita, Y., Ijiri, K., Ishidou, Y., Nagano, S., Yamamoto, T., Nagao, H., et al. (2011). Suppression of osteosarcoma cell invasion by chemotherapy is mediated by urokinase plasminogen activator activity via up-regulation of EGR1. *PLoS One* 6, e16234.

McEvilly, R. J., de Díaz, M. O., Schonemann, M. D., Hooshmand, F., and Rosenfeld, M. G. (2002). Transcriptional regulation of cortical neuron migration by POU domain factors. *Science* 295, 1528-32

McLean, C. Y., Reno, P. L., Pollen, A. A., Bassan, A. I., Capellini, T. D., Guenther, C., *et al.* (2011). Human-specific loss of regulatory DNA and the evolution of human-specific traits. *Nature* 471, 216-219.

Meyer, M., Kircher, M., Gansauge, M. T., Li, H., Racimo, F., Mallick, S., et al. (2012). A high-coverage genome sequence from an archaic Denisovan individual. *Science* 338, 222-6.

Mitchell, A. C., Jiang, Y., Peter, C., and Akbarian, S. (2015). Transcriptional regulation of GAD1 GABA synthesis gene in the prefrontal cortex of subjects with schizophrenia. *Schizophr. Res.* 167, 28-34.

Molina, V., Papiol, S., Sanz, J., Rosa, A., Arias, B., Fatjó-Vilas, M., et al. (2011). Convergent evidence of the contribution of TP53 genetic variation (Pro72Arg) to metabolic activity and white matter volume in the frontal lobe in schizophrenia patients. *Neuroimage* 56, 45-51.

Mukamel, Z., Konopka, G., Wexler, E., Osborn, G. E., Dong, H., Bergman, M. Y., et al. (2011). Regulation of MET by FOXP2, genes implicated in higher cognitive dysfunction and autism risk. *J. Neurosci.* 31, 11437-42.

Murphy, E. (2015a). The brain dynamics of linguistic computation. *Front. Psychol.* 6, 1515.

Murphy, E. (2015b). Labels, cognomes and cyclic computation: An ethological perspective. *Front. Psychol.* 6, 715.

Murphy, E. (2016). The human oscillome and its explanatory potential. *Biolinguistics* 10, 6-20.

Nakamura, T., Matsumoto, J., Takamura, Y., Ishii, Y., Sasahara, M., Ono, T, et al. (2015). Relationships among parvalbumin-immunoreactive neuron density, phase-locked gamma oscillations, and autistic/schizophrenic symptoms in PDGFR-β knock-out and control mice. *PLoS One* 10, e0119258.

Namiki, K., Nakamura, A., Furuya, M., Mizuhashi, S., Matsuo, Y., Tokuhara, N. et al. (2007). Involvement of p38alpha in kainate-induced seizure and neuronal cell damage. *J. Recept. Signal Transduct. Res.* 27, 99-111.

Narayan, S., Tang, B. Head, S. R., Gilmartin, T. J., Sutcliffe, J. G., Dean, B., et al. (2008). Molecular profiles of schizophrenia in the CNS at different stages of illness. *Brain Res.* 1239, 235-48.

Nasu, M., Yada, S., Igarashi, A., Sutoo, D., Akiyama, K., Ito, M., et al. (2014). Mammalian-specific sequences in Pou3f2 contribute to maternal behaviour. *Genome. Biol. Evol.* 6, 1145-56.

Ni, X., Trakalo, J., Valente, J., Azevedo, M. H., Pato, M. T., Pato, C. N., et al. (2005). Human p53 tumor suppressor gene (TP53) and schizophrenia: case-control and family studies. *Neurosci. Lett.* 388, 173-8.

Ohi, K., Hashimoto, R., Nakazawa, T., Okada, T., Yasuda, Y., Yamamori, H., et al. (2012).The p250GAP gene is associated with risk for schizophrenia and schizotypal personality traits. *PLoS One* 7, e35696.

Oksenberg, N., and Ahituv, N. (2013). The role of AUTS2 in neurodevelopment and human evolution. *Trends Genet.* 29, 600-8.

Oksenberg, N., Stevison, L., Wall, J. D., and Ahituv, N. (2013). Function and regulation of AUTS2, a gene implicated in autism and human evolution. *PLoS Genet.* 9, e1003221

Onwuameze, O. E., Nam, K. W., Epping, E. A., Wassink, T. H., Ziebell, S., Andreasen, N. C., et al. (2013). MAPK14 and CNR1 gene variant interactions: effects on brain volume deficits in schizophrenia patients with marijuana misuse. *Psychol. Med.* 43, 619-31.

Pääbo, S. (2014). The human condition: a molecular approach. *Cell* 157, 216-26.

Paskulin, D. d., Paixão-Côrtes, V. R., Hainaut, P., Bortolini, M. C., and Ashton-Prolla, P. (2012). The TP53 fertility network. *Genet. Mol. Biol.* 35, 939-46.

Peng, X. D., Xu, P. Z., Chen, M. L., Hahn-Windgassen, A., Skeen, J., and Jacobs, J. (2003). Dwarfism, impaired skin development, skeletal muscle atrophy, delayed bone development, and impeded adipogenesis in mice lacking Akt1 and Akt2. *Genes Dev.* 17, 1352-65.

Pérez-Santiago, J., Diez-Alarcia, R., Callado, L. F., Zhang, J. X., Chana, G., White, C. H., et al. (2012). A combined analysis of microarray gene expression studies of the human prefrontal cortex identifies genes implicated in schizophrenia. *J. Psychiatr. Res.* 46, 1464-74.

Pleasure, S. J., Anderson, S., Hevner, R., Bagri, A., Marin, O., Lowenstein, D. H., et al. (2000). Cell migration from the ganglionic eminences is required for the development of hippocampal GABAergic interneurons. *Neuron* 28, 727-40.

Potkin, S. G., Macciardi, F., Guffanti, G., Fallon, J. H., Wang, Q., Turner, J. A., et al. (2010). Identifying gene regulatory networks in schizophrenia. *Neuroimage* 53, 839-47.

Prieto del Val, L., Cantero, J. L. and Atienza, M. (2015). APOE ε4 constrains engagement of encoding-related compensatory networks in amnestic mild cognitive impairment. *Hippocampus* 25, 993-1007.

Prüfer, K., Racimo, F., Patterson, N., Jay, F., Sankararaman, S., Sawyer, S., et al. (2014). The complete genome sequence of a Neanderthal from the Altai Mountains. *Nature* 505, 43-9.

Rakhade, S. N., Shah, A. K., Agarwal, R., Yao, B., Asano, E., and Loeb, J. A. (2007). Activity-dependent gene expression correlates with interictal spiking in human neocortical epilepsy. *Epilepsia* 48 Suppl 5, 86-95.

Reale, M. E., Webb, I. C., Wang, X., Baltazar, R. M., Coolen, L. M., and Lehman, M. N. (2013). The transcription factor Runx2 is under circadian control in the suprachiasmatic nucleus and functions in the control of rhythmic behavior. *PLoS One* 8, e54317.

Roberts, J. L., Hovanes, K., Dasouki, M., Manzardo, A. M., and Butler, M. G. (2014). Chromosomal microarray analysis of consecutive individuals with autism spectrum disorders or learning disability presenting for genetic services. *Gene* 535, 70-8.

Roll, P., Rudolf, G., Pereira, S., Royer, B., Scheffer, I. E., Massacrier, A., et al. (2006). SRPX2 mutations in disorders of language cortex and cognition. *Hum. Mol. Genet.* 15, 1195-207.

Roll, P., Vernes, S. C., Bruneau, N., Cillario, J., Ponsole-Lenfant, M., Massacrier, A., et al. (2010). Molecular networks implicated in speech-related disorders: FOXP2 regulates the SRPX2/uPAR complex. *Hum. Mol. Genet.* 19, 4848-60.

Royer-Zemmour, B., Ponsole-Lenfant, M., Gara, H., Roll, P., Lévêque, C., Massacrier, A., et al. (2008). Epileptic and developmental disorders of the speech cortex: ligand/receptor interaction of wild-type and mutant SRPX2 with the plasminogen activator receptor uPAR. *Hum. Mol. Genet.* 17, 3617-30.

Ruzicka, W. B., Subburaju, S., and Benes, F. M. (2015). Circuit- and diagnosis-specific DNA methylation changes at γ-aminobutyric acid-related genes in postmortem human hippocampus in schizophrenia and bipolar disorder. *JAMA Psychiatry* 72, 541-51.

Severance, E.G., Gressitt, K.L., Stallings, C.R., Origoni, A.E., Khushalani, S., Leweke, F.M., et al. (2013). Discordant patterns of bacterial translocation markers and implications for innate immune imbalances in schizophrenia. *Schizophr. Res.* 148, 130-137.

Sollis, E., Graham, S. A., Vino, A., Froehlich, H., Vreeburg, M., Dimitropoulou, D., et al. (2016). Identification and functional characterization of de novo FOXP1 variants provides novel insights into the etiology of neurodevelopmental disorder. *Hum. Mol. Genet.* 25, 546-57.

Somel M, Liu X, and Khaitovich P. (2013). Human brain evolution: transcripts, metabolites and their regulators. *Nat. Rev. Neurosci.* 14, 112-127.

Souchet, B., Guedj, F., Sahún, I., Duchon, A., Daubigney, F., Badel, A., et al. (2014). Excitation/inhibition balance and learning are modified by Dyrk1a gene dosage. *Neurobiol. Dis.* 69, 65-75.

Španiel, F., Horáček, J., Tintěra, J., Ibrahim, I., Novák, T., Čermák, J., et al. (2011). Genetic variation in FOXP2 alters grey matter concentrations in schizophrenia patients. *Neurosci. Lett.* 493, 131-5.

Speevak, M. D. and Farrell, S. A. (2011). Non-syndromic language delay in a child with disruption in the Protocadherin11X/Y gene pair. *Am. J. Med. Genet. B Neuropsychiatr. Genet.* 156, 484-9.

Spiteri, E., Konopka, G., Coppola, G., Bomar, J., Oldham, M., Ou, J., et al. (2007). Identification of the transcriptional targets of *FOXP2*, a gene linked to speech and language, in developing human brain. *Am. J. Hum. Genet.* 81, 1144-57.

Srinivasan, S., Bettella, F., Mattingsdal, M., Wang, Y., Witoelar, A., Schork, A. J., et al. (2015). Genetic markers of human evolution are enriched in schizophrenia. *Biol. Psychiatry,* S0006-3223(15)00855-0.

St Pourcain, B., Cents, R. A., Whitehouse, A. J., Haworth, C. M., Davis, O. S., O'Reilly, P. F., et al. (2014). Common variation near ROBO2 is associated with expressive vocabulary in infancy. *Nat. Commun.* 5, 4831.

Stein, C. M., Schick, J. H., Gerry Taylor, H., Shriberg, L. D., Millard, C., Kundtz-Kluge, A., et al. (2004). Pleiotropic effects of a chromosome 3 locus on speech-sound disorder and reading. *Am. J. Hum. Genet.* 74, 283–97.

Südhof, T. C. (2008). Neuroligins and neurexins link synaptic function to cognitive disease. *Nature* 455, 903-11.

Sudmant, P. H., Kitzman, J. O., Antonacci, F., Alkan, C., Malig, M., Tsalenko, A. et al. (2010). Diversity of human copy number variation and multicopy genes. *Science* 330, 641–6.

Sugitani, M., Sugai, T., and Onoda, N. (2002). Postsynaptic activity of metabotropic glutamate receptors in the piriform cortex. *Neuroreport* 13, 1473-6.

Suntsova, M., Gogvadze, E. V., Salozhin, S., Gaifullin, N., Eroshkin, F., Dmitriev, S. E., et al. Human-specific endogenous retroviral insert serves as an enhancer for the schizophrenia-linked gene PRODH. *Proc. Natl. Acad. Sci. U.S.A.* 110, 19472-7.

Szklarczyk, D., Franceschini, A., Wyder, S., Forslund, K., Heller, D., Huerta-Cepas, J., et al. (2015). STRING v10: protein-protein interaction networks, integrated over the tree of life. *Nucleic Acids Res.* 43, D447-52.

Thygesen, J. H., Zambach, S. K., Ingason, A., Lundin, P., Hansen, T., Bertalan, M. et al. (2015). Linkage and whole genome sequencing identify a locus on 6q25-26 for formal thought disorder and implicate MEF2A regulation. *Schizophr. Res.* 169, 441-6.

van Bon, B. W., Balciuniene, J., Fruhman, G., Nagamani, S. C., Broome, D. L., Cameron, E., et al. (2011). The. phenotype. of. recurrent. 10q22q23 deletions and duplications. *Eur. J. Hum. Genet.* 19, 400-8.

Vawter, M. P., Usen, N., Thatcher, L., Ladenheim, B., Zhang, P., VanderPutten, D. M., et al. (2001). Characterization of human cleaved N-CAM and association with schizophrenia. *Exp. Neurol.* 172, 29-46.

Verbrugghe, P., Bouwer, S., Wiltshire, S., Carter, K., Chandler, D., Cooper, M., et al. (2012). Impact of the Reelin signaling cascade (ligands-receptors-adaptor complex) on cognition in schizophrenia. *Am. J. Med. Genet B Neuropsychiatr. Genet.* 159B, 392-404.

Veyrac, A., Besnard, A., Caboche, J., Davis, S., and Laroche S. (2014). The transcription factor Zif268/Egr1, brain plasticity, and memory. *Prog. Mol. Biol. Transl. Sci.*122, 89-129.

Waltereit, R., Leimer, U., von Bohlen und Halbach, O., Panke, J., Hölter, S. M., Garrett, L., et al. (2012). Srgap3^{-}/$^{-}$ mice present a neurodevelopmental disorder with schizophrenia-related intermediate phenotypes. *FASEB J.* 26, 4418-28.

Wang, R. (2011). Dissecting the Genetic Basis of Convergent Complex Traits Based on Molecular Homoplasy. PhD, Duke University.

Wang, Y., Huang, Y., Peng, M., Cong, Z., Li, X., Lin, A., et al. (2015). Association between Silent Information Regulator 1 (SIRT1) gene polymorphisms and schizophrenia in a Chinese Han population. *Psychiatry Res.* 225, 744-5.

Willemsen, M. H., Nijhof, B., Fenckova, M., Nillesen, W. M., Bongers, E. M., Castells-Nobau A, et al. (2013). GATAD2B loss-of-function mutations cause a recognisable syndrome with intellectual disability and are associated with learning deficits and synaptic undergrowth in Drosophila. *J. Med. Genet.* 50, 507-14.

Williams, N. A., Close, J. P., Giouzeli, M., and Crow, T. J. (2006). Accelerated evolution of Protocadherin11X/Y: A candidate gene-pair for cerebral asymmetry and language. *Am. J. Med. Genet. B Neuropsychiatr. Genet.* 141, 623-33.

Wilson, N. K., Lee, Y., Long, R., Hermetz, K., Rudd, M. K., Miller, R., et al. (2011). A novel microduplication in the neurodevelopmental gene SRGAP3 that segregates with psychotic illness in the family of a COS proband. *Case Rep. Genet.* 2011, 585893.

Wockner, L. F., Noble, E. P., Lawford, B. R., Young, R. M., Morris, C. P., Whitehall, V. L., et al. (2014). Genome-wide DNA methylation analysis of human brain tissue from schizophrenia patients. *Transl. Psychiatry* 4, e339.

Yamada, K., Iwayama, Y., Hattori, E., Iwamoto, K., Toyota, T., Ohnishi, T., et al. (2011). Genome-wide association study of schizophrenia in Japanese population. *PLoS One* 6, e20468.

Yolken, R.H., Severance, E.G., Sabunciyan, S., Gressitt, K.L., Chen, O., Stallings, C., et al. (2015). Metagenomic sequencing indicates that the oropharyngeal phageome of individuals with schizophrenia differs from that of controls. *Schizophr. Bull.* 41, 1153-1161.

Zhang, B., Xu, Y. H., Wei, S. G., Zhang, H. B., Fu, D. K., Feng, Z. F., et al. (2014). Association study identifying a new susceptibility gene (AUTS2) for schizophrenia. *Int. J. Mol. Sci.* 15, 19406-16.

Zhang, J., Liao, G., Liu, C., Sun, L., Liu, Y., Wang, Y., et al. (2011). The association of CLOCK gene T3111C polymorphism and hPER3 gene 54-nucleotide repeat polymorphism with Chinese Han people schizophrenics. *Mol. Biol Rep.* 38, 349-54.

Zweier, C. (2012). Severe intellectual disability associated with recessive defects in CNTNAP2 and NRXN1. *Mol. Syndromol.* 2, 181-185.
